# The role of intrasexual competition on the evolution of male-male courtship display: a systematic review

**DOI:** 10.7717/peerj.14638

**Published:** 2023-02-02

**Authors:** Inês Órfão, Constança Carvalho, Inês Rodrigues, Leonor Ascensão, Marie Pedaccini, Luís Vicente, Miguel Barbosa, Susana A. M. Varela

**Affiliations:** 1 CFCUL–Centre for Philosophy of Sciences of the University of Lisbon, Faculty of Sciences of the University of Lisbon, Lisboa, Portugal; 2 cE3c–Centre for Ecology, Evolution and Environmental Changes, Faculty of Sciences of the University of Lisbon, Lisboa, Portugal; 3 MARE–Marine and Environmental Sciences Centre/ARNET–Aquatic Research Network, Agência Regional para o Desenvolvimento da Investigação Tecnologia e Inovação (ARDITI), Funchal, Madeira, Portugal; 4 ISPA–Instituto Universitário, Lisboa, Portugal, Lisboa, Portugal; 5 Faculty of Sciences of the University of Lisbon, Lisboa, Portugal; 6 Ghent University, Ghent, Belgium; 7 Department of Animal Biology, Faculty of Sciences of the University of Lisbon, Lisboa, Portugal; 8 School of Psychology and Life Sciences of the Lusófona University, Lisboa, Portugal; 9 School of Biology, University of St Andrews, Centre for Biological Diversity, St Andrews, United Kingdom; 10 CESAM–Centro de Estudos do Ambiente e do Mar, Departamento de Biologia, Universidade de Aveiro, Aveiro, Portugal; 11 IGC–Instituto Gulbenkian de Ciência, Oeiras, Portugal; 12 William James Center for Research, ISPA–Instituto Universitário, Lisboa, Portugal

**Keywords:** Sexual selection, Intrasexual selection, Communication network, Sexual information, Sexual signals’ dual function, Trait co-option, Display, Social information

## Abstract

**Background:**

Evidence of male-male courtship display is widespread across the animal kingdom. Yet, its function and evolutionary origin remain unclear. Here, we hypothesise that male-male courtship display evolved in response to selection pressure exerted by intrasexual competition during male-female courtship interactions. Intrasexual competition can be caused by bystander male pressure through eavesdropping and exploiting on displayer male’s courtship interactions with females. This bystander pressure can lead to an audience effect by the displayer, who will change their courtship behaviour in the presence of bystanders and display directly towards them, even in the absence of females, as an intimidation strategy. In species where this selection pressure has taken place, we predict that the male courtship display will have a dual function: attract females and deter competitors. Therefore, we expected to find more evidence of bystander-related behaviours in species for which male-male courtship display is linked to intrasexual competition compared to species for which other explanatory hypotheses are more plausible (*e.g*., mistaken identity or courtship practice).

**Methodology:**

We conducted two systematic reviews to test this hypothesis. First, we conducted a search for studies of species with courtship display between males and of the hypotheses provided to explain this behaviour. Our goal was to identify the species with male-male courtship display and evidence of intrasexual competition. Second, among the species with male-male courtship display, we searched for evidence of bystander-related behaviours, *i.e*., articles referring to eavesdropping, exploitation, and audience effect during male-female courtship interactions. Our goal was to test whether species with intrasexual competition are also more likely to show bystander-related behaviours.

**Results:**

Although most studies reporting male courtship display towards other males do not suggest any explanatory hypothesis for this behaviour, the intrasexual competition hypothesis was largely mentioned and supported by some studies reviewed. Additionally, there is more evidence of eavesdropping and of all three bystander-related behaviours combined in species for which the intrasexual competition hypothesis was suggested.

**Conclusions:**

Overall, our review supports the hypothesis that intrasexual competition can play a key role in male courtship display evolution, namely that male-male courtship display may have evolved as a secondary function of male-female courtship interactions *via* bystander male pressure. However, our review also shows that despite the increasing interest in same-sex sexual behaviours, and male-male courtship display in particular, most studies were found to be merely descriptive, and the hypotheses they suggested to explain courtship display between males mostly speculative. This highlights an important gap in the literature. To clarify both the evolution and the function of male-male courtship display, this behaviour needs to be empirically studied more often. Our review can help advancing this research area, as it makes the 20 species with male-male courtship display for which the intrasexual competition hypothesis was suggested excellent candidates for empirical research.

## Introduction

Sexual behaviours directed at individuals of the same sex—generally termed “same-sex sexual behaviour”—have generated increasing interest in recent decades, with records in all major vertebrate clades and in many invertebrate groups (Bailey & Zuk, 2009; Monk et al., 2019; Roughgarden, 2004; Balfour & Shuker, 2020; Scharf & Martin, 2013). Male-male courtship display, which occurs when males display towards other males the same way they display to females while courting, is one common same-sex sexual behaviour (see Glossary). While several hypotheses have been proposed to explain male-male courtship display (Table 1), its evolutionary history remains poorly understood, as for same-sex sexual behaviours in general (Bailey & Zuk, 2009; Monk et al., 2019). Some hypotheses propose maladaptive explanations, such as misidentifications of the opposite sex, while others see an adaptive value in this behaviour (Bailey & Zuk, 2009; Monk et al., 2019). One of the latter is the intrasexual competition hypothesis—also named “intrasexual conflict” by Bailey & Zuk (2009). According to this hypothesis, male courtship display towards other male can be used to establish dominance or inhibit aggression, among other advantages (see Table 1). Here, we use systematic reviews to study the likelihood of intrasexual competition in the evolution of male-male courtship display.

**Scheme 1:** Glossary.

Same-sex sexual behaviour	Sexual behaviours-such as courtship, mounting, genital contact, pair bonding and offspring raising-directed to individuals of the same sex.
Courtship display	Conspicuous behaviour, often performed by males, with the function of attracting mates, promoting the reproductive success of the displayer. This behaviour is usually performed with the help of ornaments—such as bright colours or conspicuous appendages (*e.g*., crowns and tails)—or vocalizations, to enhance these secondary sexual traits. In some cases, ornaments may be features that extend the physical phenotype of males (Dawkins, 1989). For example, bower constructions by satin bowerbird males *Ptilonorhynchus violaceus* influence female choice (Borgia, 1985).
Displayer	Any individual that performs a conspicuous behaviour directed to or intercepted by one or more individuals (*i.e*., audience). In this study we consider males as the displayer individuals during courtship interactions.
Male-male courtship display (MMCD)	Any displaying behaviour performed by a male towards other males, exactly as males would display towards females during a mating context. However, displayers are not necessarily courting (*i.e*., attracting) other males. If this was always the case, then MMCD would be always mal-adaptive, which opposes some evidence supporting that it contributes to male fitness if its function is to intimidate rivals (Bierbach et al., 2013; Steiner, Steidle & Ruther, 2005). MMCD does not include male displaying behaviours exclusively directed towards other males during an agonistic context, such as agonistic displays performed by *Psolodesmus* sp. damselfly males towards intruders (Batucan et al., 2021).
Sexual signal dual function	Secondary sexual traits—such as ornaments and armaments—used both in male-male competitive and male-female courtship interactions.
Bystander	Any individual that is not actively and directly involved in a social interaction but observes and extracts information from that interaction (see eavesdropping). In this study we consider as bystanders, males that eavesdrop on other males’ courtship interactions with females.
Bystander-related behaviours (BRB)	Actions or interactions performed or influenced by a bystander, such as eavesdropping, exploitation, and audience effect.
Eavesdropping	When a bystander extracts information from the actions or interactions of other individuals in which he is not directly involved (Wiley, 1983; McGregor, 1993; McGregor & Dabelsteen, 1996; McGregor & Peake, 2000; McGregor, 2005; Danchin, Giraldeau & Wagner, 2008).
Exploitation	When a bystander eavesdrops on another male courtship interactions with females and uses the extracted information to his own benefit leading to direct costs to the displayer (McGregor & Dabelsteen, 1996; Danchin, Giraldeau & Wagner, 2008).
Audience effect	When a displayer is aware of the presence of a bystander and changes his behaviour accordingly (Marler & Evans, 1996; Danchin et al., 2004; Danchin, Giraldeau & Wagner, 2008). This behavioural change may include deception, *i.e*., when individuals manipulate the behaviour of others by transmitting non-reliable information by lying, withholding information, attenuating, bluffing, or exaggerating (Bradbury & Vehrencamp 2000; Searcy & Nowicki, 2005).

**Table 1:** Hypotheses suggested to explain male-male courtship display.

Hypotheses		Sub-hypothesis	Description
**Adaptive value**			
	Competition	Defence	Used to defend a territory or resource (food or female)
		Intimidation	Used to obtain a resource
		Aggressiveness inhibitor	Inhibits aggression of competitor males
		High competition	Inhibits aggression in environments where male-male encounters are frequent
		Sexual interference	Takes place during a mating attempt of another male eventually stopping it
		Dominance hierarchy	Helps to define or maintain a hierarchical position
		Condition assessment	Allows to assess the phenotypic condition of a competitor male
	Social glue		Promotes affiliative relationships between males
	Practice		Unexperienced juveniles learn from adult males
**Non-adaptive value**			
	Displaced or abnormal behaviour	Isolation or no opportunity to mate (prison effect)	When males have no access to females for a certain period
		High competition environment	When males face a high rate of encounters with another males
		Mutations	When induced (laboratory) or natural (rare) mutations change males’ sexual behaviours
	Byproduct	Hibernation	When males perform this behaviour immediately after stopping hibernation
		Sexual motivation	High male libido after previous encounters with females
	Mistaken identity		When males do not distinguish other males from females
**Not specified (possibly adaptive)**			
	Early experience	Isolation or no opportunity to mate	When males have no access to females for a certain period
		High competition environment	When males face a high rate of encounters with another males
	Sexual preference		When males can choose between females and males, but prefer males
	Thermoregulation		Increase the body temperature of males protecting them

**Note:**

Hypotheses based on review studies on same-sex sexual behaviours and studies on male-male courtship display (Bailey & Zuk, 2009; Balfour & Shuker, 2020; Monk et al., 2019; Scharf & Martin, 2013; and references in Table S1).

Male courtship display is a sexual signal that conveys information about the motivation, condition and/or quality of displayer males, which both females and male competitors can use for decision-making (Andersson, 1994; Andersson & Simmons, 2006; Chandler, Ofria & Dworkin, 2012; Kuijper, Pen & Weissing, 2012; see Glossary). For instance, a male displaying to a female can inform a bystander of the displayer’s motivation to mate and his willingness to compete for mating. Also, male courtship display can inform other males about a female’s location, quality, or sexual receptiveness (Milner, Jennions & Backwell, 2010; Auld, Jeswiet & Godin, 2015; Uetz et al., 2019). Additionally, female response to male courtship can provide information to bystanders about the displayer’s quality and condition and, hence, his ability to compete (Clark, Roberts & Uetz, 2012; Garcia et al., 2019). Consequently, the bystander can use courtship display to detect a female, decide whether to court her and how much to invest (Murai, Koga & Yong, 2002; Clark, Roberts & Uetz, 2012; Zhang et al., 2016). The displayer, in turn, can change the features of his courtship display to reduce the risk of competition. For example, the displayer can decrease the intensity of his display to reduce conspicuousness. Alternatively, he can increase display intensity to reinforce intimidation (Earley, 2010; Plath & Bierbach, 2011; Castellano, Friard & Pilastro, 2016; Zhang et al., 2016). Males can, therefore, influence their competitors’ mating strategies while displaying to females, as well as being influenced by their competitors’ presence and behaviour. This suggests that the male courtship display can have a dual function—attracting females and deterring rivals—and, consequently, that male-male courtship display may have originated from intrasexual competition.

The dual function of male secondary sexual traits (expressed as visual, chemical, electric, and acoustic signals) is well documented (see Glossary and reviews in Searcy & Andersson, 1986; Berglund, Bisazza & Pilastro, 1996; Borgia, 2006). It is also commonly suggested that sexual signals evolved as armaments through intrasexual selection and were later co-opted as ornaments in female choice (Berglund, Bisazza & Pilastro, 1996). This seems to be the case with the evolution of antlers in the white-tailed male deer *Odocoileus virginianus*. In this species, the size of antlers provides benefits in a male-male competition context but are later used by females to assess male quality (Berglund, Bisazza & Pilastro, 1996; Morina et al., 2018). Further, in the gregarious cricket *Amphiacusta maya* courtship chirps are primarily used to warn other males and secondly to increase receptivity in females (Boake & Capranica, 1982). Other studies, however, have suggested a primary role of female choice in the evolution of male secondary sexual traits. For example, the pigmented vertical bars in northern swordtail fish *Xiphophorus* spp. evolved to attract females and were later co-opted to deter aggression from competitor males (Morris, Tudor & Dubois, 2007). The boatwhistles in the Lusitanian toadfish *Halobatrachus didactylus* are mainly used as an ornament but also signal territorial ownership (Vasconcelos et al., 2010). The courtship display is another type of sexual trait expressed as body movements or dances, which often also serves to highlight other ornaments, such as sizes, colours and vocalizations (*e.g*., Oliveira & Custodio, 1998; Kodric-Brown & Nicoletto, 2001; Borgia & Coleman, 2000; respectively). However, despite the relevance of courtship for mate choice and competition, and co-option being an important mechanism for the evolution of male sexual traits (Borgia, 2006), few studies have examined the dual function of the courtship display (Pope, 2000; Delaney, Roberts & Uetz, 2007; Yorzinski et al., 2017).

**Figure 1:**
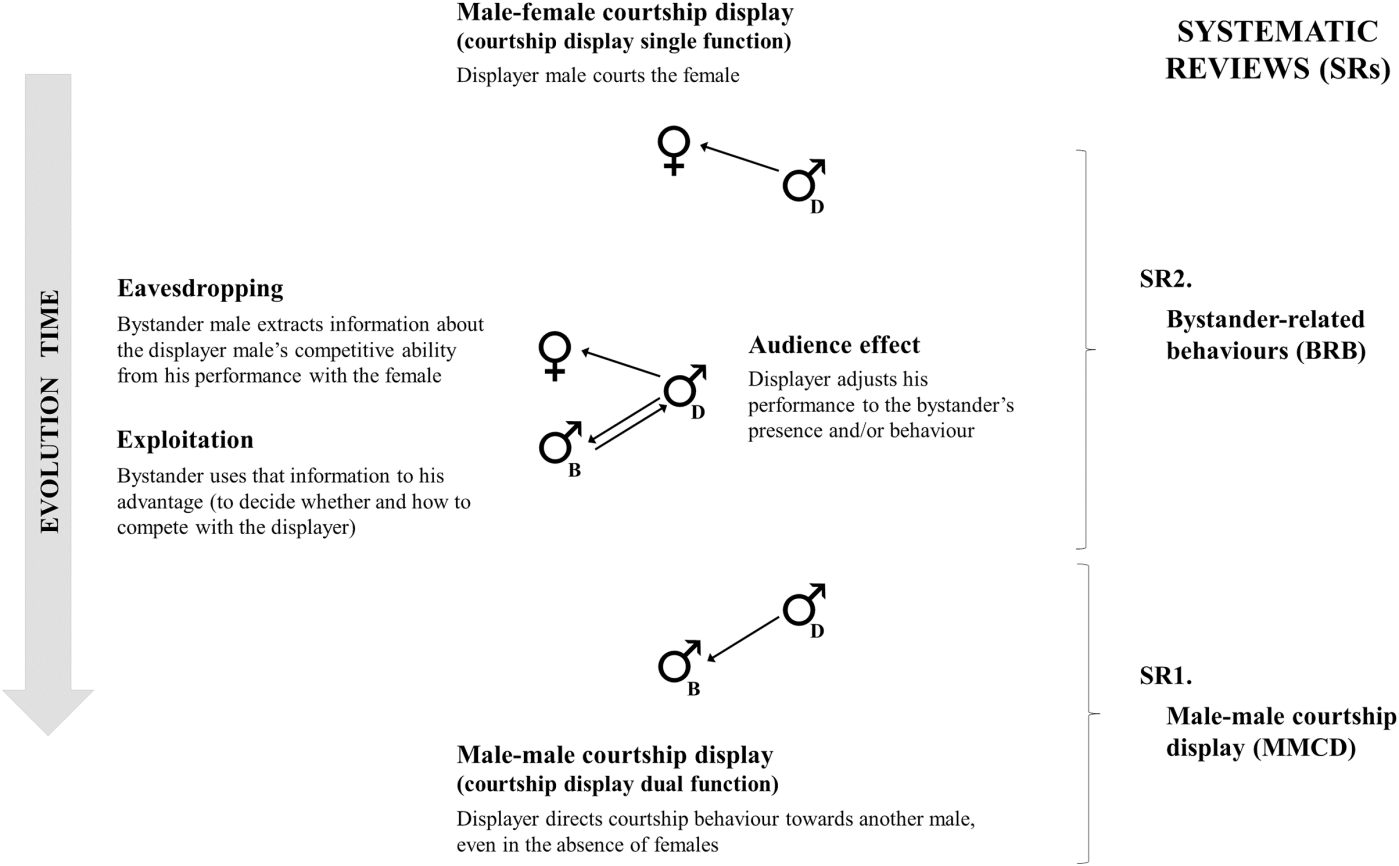
Possible evolutionary stages of male-male courtship display. Possible evolutionary stages of male-male courtship display (MMCD; on the left) and systematic reviews performed (SR; on the right). Proposed evolutionary steps start with male-female courtship display (courtship display has a single function, in the top), followed by bystander-related behaviours (BRB: eavesdropping and exploitation by the bystander—represented with “B”—and audience effect by the displayer male—represented with “D”). Finally, courtship display gains a dual function with the appearance of MMCD (in the bottom). The systematic reviews focused on (from bottom to top): (1) MMCD and the hypotheses suggested to explain this behaviour (SR1), and (2) bystander-related behaviours during male-female courtship display (SR2).

In this study, we investigate the evolution of male-male courtship display as the result of intrasexual competition leading to a dual function signal. We propose the co-option of male-female courtship interactions to male-male competitive interactions. The presence or interference of competitor males in male-female courtship interactions may have been the selection pressure that favoured the emergence of male-male courtship display, with possibly five evolutionary stages (Fig. 1; see Glossary). First, courtship is a signal with a single function that males use to attract females. Then, bystander males may become part of this interaction being also attracted to other males’ courtship interactions with females and extract information about the presence or quality of females, and/or about the competitive ability of displayers (eavesdropping; Wiley, 1983; McGregor, 1993; Danchin, Giraldeau & Wagner, 2008). Additionally, bystanders can use this information to their advantage (exploitation; McGregor, 2005). For example, to initiate a territorial fight with the displayer if of inferior quality. As a response to this bystander pressure, displayers can adjust their behaviour (audience effect; Matos & Schlupp, 2005; Kniel, Bender & Witte, 2016), by reducing, exaggerating, or matching the intensity and frequency of their display to the bystander (*e.g*., Vignal, Mathevon & Mottin, 2004; Fisher & Rosenthal, 2007; Auld & Godin, 2015). Lastly, males can direct courtship towards competitors—even in the absence of females—as a way of defending their territory or discouraging competitors from displaying to nearby females or starting a fight. At this evolutionary stage the courtship display classifies as same sex sexual behaviour.

To study the likelihood of courtship display directed to males as the result of bystander pressure, we conducted two systematic reviews: (1) to identify all species with evidence of male-male courtship display for which the hypothesis of intrasexual competition has been proposed and/or tested, and (2) to find evidence of bystander-related behaviours (eavesdropping, exploitation, and audience effect) during male-female courtship interactions within the previously listed species. Our results corroborate the hypothesis that species for which male-male courtship display is described to have a competitive function are also species for which there is more evidence of bystander pressure.

## Survey methodology

**Figure 2:**
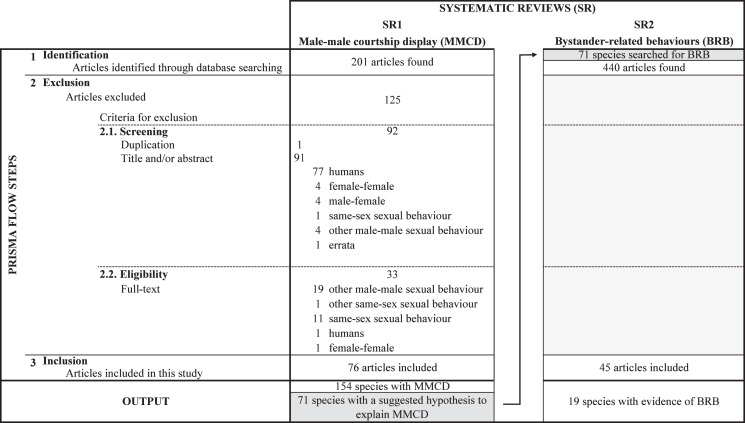
Flow diagram with search results for the two systematic reviews (SRs). Flow diagram with search results for the two systematic reviews (SRs). The SRs were based on the PRISMA flow by Moher et al. (2009), in which: (1) all possible articles were searched based on search criteria (“identification” step), (2) non-relevant articles were removed (“exclusion” step), and (3) articles with relevant information for posterior analysis were included (“inclusion” step). In the first systematic review (SR1), we searched for species with male-male courtship display (MCCD) and the hypotheses suggested to explain this behaviour. This SR1 followed exactly the four steps of the PRISMA flow, and the exclusion process was split into “screening” (2.1.) and “eligibility” (2.2.), *i.e*., articles were first excluded based on title and/or abstract and, later, based on the full text. Reasons for exclusion are given in the figure. We found 76 articles describing 154 species with evidence of MMCD, 71 with at least one hypothesis about the evolution and/or function of this behaviour. In the second systematic review (SR2), we searched for bystander-related behaviours (BRB) for each of these 71 species. This SR2 followed only two steps of the PRISMA flow: identification (1) and inclusion (2). When an article mentioned a searched BRB (*i.e*., eavesdropping, exploitation, or audience effect) during male-female courtship interactions, we stopped the search for that BRB. Otherwise, we evaluated the next article and, so on, until finding any evidence or until finding no evidence for that BRB in all identified articles. The inclusion process was based on the articles’ full text. We found 45 articles with evidence of BRB for 19 of the 71 species.

We used the Web of Science to perform the two systematic reviews (Fig. 2; SR1 and SR2). Systematic reviews were carried out between April 2016 and May 2018 and were updated in January 2022. We searched for keywords in the field “Topic (TS)”, which included articles’ title, abstract and keywords, as well as indexing fields such as systematics, taxonomic terms and descriptors, and in “keywords plus” (keywords added by “Thomson Reuters editorial expertise in science”). Our search terms retrieved both British and American English spelling, and related expressions. For example, by searching “behavio*” we could find articles mentioning “behavior”, “behaviour”, “behaviors” or “behaviours”. This made our search comprehensive, increasing the likelihood of finding all relevant papers.

### Male-male courtship display

In the first systematic review we quantified the studies, the species and the hypotheses put forward to explain male-male courtship display. To achieve this goal, we included generalist terms in the search to widen the probability of capturing the most relevant studies. Specifically, we searched for articles with: “same-sex sexual behavio*” OR “same-sex sexual display” OR “same-sex courtship” OR “sexual display toward? *other male” OR “male-male sexual display” OR “male-male courtship” OR “male-male sexual behavio*” OR “sexual display between males” OR “sexual behavio* between males”. We restricted the search to specific research areas because we were only interested in non-human animal studies. Therefore, the search was conducted within the following research areas: “behavioural sciences or psychology or zoology or evolutionary biology or reproductive biology or sociology or marine freshwater biology or environmental sciences ecology or social sciences other topics or science technology other topics or entomology or social issues or fisheries or communication”. Two independent raters (IR and LA, or IR and IO) screened all articles found (*n* = 201; see SR1 in Fig. 2). The two raters selected articles independently and recorded whether each article mentioned or described male-male courtship display. The two lists were then compared by a third person (IO or MP) to solve any potential disagreement between the two raters on study selection. From the initial 201 potential studies, 76 articles were deemed to meet the inclusion criteria (see References S1).

From this list of 76 articles, we identified 154 species with male-male courtship display (see Table S1), as well as the hypotheses used to explain the evolution and/or function of this behaviour for each species (following Table 1). The hypotheses were classified into three categories: ‘competition’, ‘other’ (any other hypotheses but competition—*e.g*., ‘mistaken identity’), and ‘none’ (no hypothesis suggested) (see Table S1). A species was categorised under ‘competition’ when the study suggested the intrasexual competition hypothesis as a possible explanation for male-male courtship display. If the study suggested several hypotheses in addition to intrasexual competition, the species was also included in the ‘competition’ category unless the study found evidence against it (*e.g*., in the spring field cricket *Gryllus veletis*, Boutin et al., 2016). On the other hand, a species was categorised under ‘other’ when one or multiple hypotheses other than intrasexual competition were given. Additionally, the ‘none’ category included species for which studies did not mention any hypothesis, or argued against one hypothesis but did not suggest an alternative one. Hypothesis categorization was performed by two independent raters (IO and CC, or IO and MP), whose scores were compared to dismiss disagreements (see Table S1 describing all hypotheses listed by species). There was 87.7% of inter-raters agreement (229 agreements out of 261 times that male-male competition was mentioned for the 154 species). When the two independent raters disagreed, a consensus was reached after a detailed discussion. We found 20 species for the ‘competition’ category, 51 for ‘other’ and 83 for ‘none’.

### Bystander-related behaviours

In the second systematic review, we collected evidence for bystander-related behaviours during male-female courtship for the species listed in the first systematic review. Only species for which an evolutionary explanation has been provided were considered (*n* = 71), meaning that we excluded the 83 species of the ‘none’ category. Based on bystander pressure as the possible evolutionary mechanism for male-male courtship display we predicted that bystander-related behaviours such as eavesdropping, exploitation, and audience effect, as well as the three behaviours combined, would be more prevalent in species where the intrasexual competition hypothesis was suggested. For each of the 71 species, we searched articles including the species’ scientific or common name, and the following search terms: (“sexual behavio*” OR “sexual display” OR “court*”) AND (“conspecific” OR “observer” OR “public” OR “viewer” OR “spectator” OR “eavesdrop*” OR “bystander” OR “receiver” OR “presence” OR “audience” OR “exploit*”). As previously, we used general terms to increase the probability of finding most articles (*e.g*., “sexual behaviour” to find male courtship display and “conspecific” to find bystander-related behaviours—see Glossary). We found 440 studies in total (an average of 6 studies per species), which were subsequently screened by two independent raters (IO and CC, or IO and MP; Fig. 2).

The review process was carried out for each species as follows: we looked for evidence of bystander-related behaviours in full text from the most recent to oldest articles and stopped the search when evidence of the three bystander-related behaviours was found. If no articles were left to be analysed, we assumed there was no evidence of bystander pressure for that species. Three articles were excluded from the analyses since the evidence supporting the authors’ suggestion of bystander-related behaviours was unclear. This systematic review included 45 articles and 19 species with evidence of bystander-related behaviours (see SR2 in Fig. 2 and References S2).

### Statistical analyses

The data collected from the second systematic review were statistically analysed. We used Fisher’s exact tests of independence to see whether the species for which the intrasexual competition hypothesis has been suggested are also the species for which there is more evidence of bystander-related behaviours. Fisher’s exact is suitable for the collected data with small sample sizes that do not fit the assumption of chi-squared test—*i.e*., expected frequencies are less than 5 in more than 20% of the cells. We created 2 × 2 contingency tables for two nominal variables, which included the number of species per suggested male-male courtship display hypothesis (‘competition’ *vs*. ‘other’) and evidence of bystander-related behaviour (‘evidence’ *vs*. ‘no-evidence’). We analysed each bystander-related behaviour (eavesdropping, exploitation, and audience effect) separately and combined. We repeated the same analyses after removing species from the ‘competition’ category for which more than one hypothesis, in addition to intrasexual competition, has been suggested to ensure that results are not due to a confounding effect caused by the suggestion of multiple explanatory hypotheses. All analyses were performed using R 4.1.1. (R Core Team, 2020). Values were considered statistically significant when *p* ≤ 0.05.

## Survey results

### Male-male courtship display

**Figure 3:**
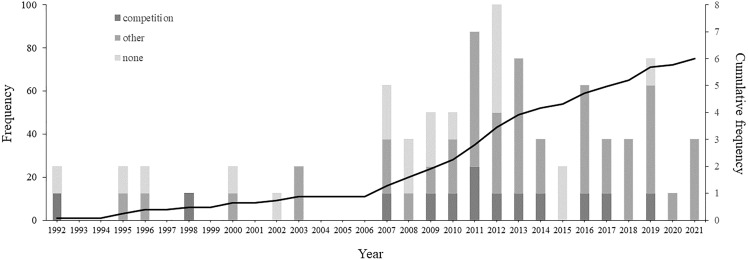
Temporal distribution of articles referring the male-male courtship display on non-human animals. Barplots represent the frequency of articles per year of publication, where the number of articles that suggested any or no explanatory hypothesis are represented by different colours: darker grey indicates articles that suggested the intrasexual competition hypothesis, intermediate grey indicates articles that suggest other hypotheses than intrasexual competition, and lighter grey indicate articles that did not suggested any hypothesis. The line across the bars represents the cumulative frequency.

There has been an increase in the number of studies describing courtship between males in non-human animals from 1992 to 2021 (Fig. 3). The 76 articles that describe male-male courtship display identify this behaviour in 154 species belonging to six Classes (see Table S1): Insecta (67 species, 43.5%), Aves (55 species, 35.7%), Mammalia (25 species, 16.2%), Actinopterygii (three species, 1.9%), Arachnida (two species, 1.3%), and Reptilia (two species, 1.3%).

No explanatory hypothesis was suggested for more than half the species (83 species, 53.9%; see Fig. 3 and Table S1). When at least one hypothesis was suggested (71 species, 46.1%), intrasexual competition was indicated for 20 species (13.0%), belonging to five Classes: Reptilia (one species, 5.0%), Actinopterygii (one species 5.0%), Mammalia (three species, 15.0%), Aves (seven species, 35.0%) and Insecta (eight species, 40.0%).

From the 14 studies that mentioned competition, most are from the past two decades (92.9%; see Fig. 3 and Table 2). Three studies were literature reviews on same-sex sexual behaviour (Bailey & Zuk, 2009; MacFarlane, Blomberg & Vasey, 2010; Scharf & Martin, 2013), and eleven were experimental studies. However, only five of the experimental studies explicitly tested hypotheses for male-male courtship display. Four of the studies tested the intrasexual competition hypothesis (Boutin et al., 2016; Lane et al., 2016; Kuriwada, 2017; Rayner & Bailey, 2019), and two found evidence to support it (Lane et al., 2016; Kuriwada, 2017). Further, a single study tested another hypothesis but ended up suggesting competition (Abbassi & Burley, 2012).

**Table 2:** Articles referring the male-male courtship display and the competition hypothesis on non-human animals.

Type	(Sub) Hypothesis	Testing	Evidence	Class	Species	References
Review	Competition in general	na	na	in general		Bailey & Zuk (2009)
		na	na	Reptilia	*Thamnophis sirtalis parietalis*	Bailey & Zuk (2009)
		na	na	Aves	in general	MacFarlane, Blomberg & Vasey (2010)
		na	na	Aves	*Cygnus atratus*	MacFarlane, Blomberg & Vasey (2010)
		na	na	Aves	*Melanerpes formicivorus*	MacFarlane, Blomberg & Vasey (2010)
		na	na	Aves	*Ptilonorhynchus violaceus*	MacFarlane, Blomberg & Vasey (2010)
		na	na	Aves	*Rupicola rupicola*	MacFarlane, Blomberg & Vasey (2010)
		na	na	Insecta	in general	Scharf & Martin (2013)
		na	na	Insecta	flies and wasps	Scharf & Martin (2013)
		na	na	Insecta	*Aphidius ervi*	Scharf & Martin (2013)
		na	na	Insecta	*Byrsotria fumigata*	Scharf & Martin (2013)
		na	na	Insecta	*Cephalonomia tarsalis*	Scharf & Martin (2013)
		na	na	Insecta	*Cotesia rubecula*	Scharf & Martin (2013)
		na	na	Insecta	*Euphydryas editha*	Scharf & Martin (2013)
		na	na	Insecta	*Eupoecilia ambiguella*	Scharf & Martin (2013)
Empirical	Competition as				* *	
	Dominance hierarchy	−	−	Actinopterygii	*Oreochromis mossambicus*	Oliveira & Almada (1998)
		−	−	Mammalia	*Capra hircus*	Ungerfeld et al. (2014)
		−	−	Mammalia	*Ovis aries*	Ungerfeld, Ramos & Bielli (2007)
		−	−	Mammalia	*Ovis canadensis*	Ungerfeld, Ramos & Bielli (2007)
		−	−	Insecta	in general	Wang et al. (2011)
		−	−	Aves	*Poephila acuticauda*	Adachi & Soma (2019)
		+	−	Insecta	*Gryllus veletis*	Boutin et al. (2016)
	Condition assessment	+	+	Aves	*Melopsittacus undulatus*	Abbassi & Burley (2012)
	Resource defence	+	+	Aves	*Melopsittacus undulatus*	Abbassi & Burley (2012)
		−	−	Aves	*Taeniopygia guttata*	Elie, Mathevon & Vignal (2011)
	Aggressiveness inhibitor	+	+	Insecta	*Gnatocerus cornutus*	Lane et al. (2016)
		+	+	Insecta	*Teleogryllus occipitalis*	Kuriwada (2017)
		+	−	Insecta	*Teleogryllus oceanicus*	Rayner & Bailey (2019)

**Note:**

Articles referring to male-male courtship display and the intrasexual competition hypothesis on non-human animals. Type of article, sub-hypothesis suggested, whether the article tested an explanatory hypothesis or nor (represented as “+” or as “−”, respectively), and whether there was evidence supporting the intrasexual competition hypothesis (represented as “+” or as “−”, respectively), year of publication and authors.

Different mechanisms of intrasexual competition were suggested in the experimental studies (Table 2): male-male courtship display can be relevant for “resource defence” (Elie, Mathevon & Vignal, 2011; Abbassi & Burley, 2012); it plays a role on “condition assessment” of the displayer or its opponents (Abbassi & Burley, 2012); and males assess the displayer condition to modulate aggressiveness (Lane et al., 2016; Kuriwada, 2017) by “diverting costly aggression” (Rayner & Bailey, 2019), or to establish “dominance hierarchies” (Oliveira & Almada, 1998; Ungerfeld, Ramos & Bielli, 2007; Wang et al., 2011; Ungerfeld et al., 2014); with dominant males performing more male-male courtship than subordinate ones (Adachi & Soma, 2019 referring to Langmore & Bennett, 1999).

Half species with male-male courtship display linked to competition have been also credited with another explanatory hypothesis (Table 3). For eight species only one other hypothesis was suggested, while for two species two other hypotheses were proposed. The mistaken identity hypothesis, a non-adaptive explanation, was suggested by the same study for five species (Scharf & Martin, 2013). The other hypotheses were only mentioned once by the same or different studies: three studies suggested other non-adaptive explanations, such as displaced behaviour (Oliveira & Almada, 1998; Amorim, Fonseca & Almada, 2003; Ungerfeld et al., 2014) and three studies suggested adaptive explanations, such as social glue and practice (MacFarlane, Blomberg & Vasey, 2010), and thermoregulation (Bailey & Zuk, 2009; Shine et al., 2000).

**Table 3:** Species for which the intrasexual competition hypothesis has been suggested to explain male-male courtship display (MMCD), either exclusively or simultaneously with other hypotheses. Hypotheses, species’ Class and scientific name, and supporting references for the suggested hypothesis.

Hypotheses MMCD			Class	Species	References
Competition			Aves	*Melanerpes formicivorus*	MacFarlane, Blomberg & Vasey (2010)
			Aves	*Melopsittacus undulatus*	Abbassi & Burley (2012)
			Aves	*Poephila acuticauda*	Adachi & Soma (2019)
			Aves	*Rupicola rupicola*	MacFarlane, Blomberg & Vasey (2010)
			Aves	*Taeniopygia guttata*	Elie, Mathevon & Vignal (2011)
			Insecta	*Cephalonomia tarsalis*	Scharf & Martin (2013)
			Insecta	*Gnatocerus cornutus*	Lane et al. (2016)
			Insecta	*Teleogryllus occipitalis*	Kuriwada (2017)
			Mammalia	*Ovis aries*	Ungerfeld, Ramos & Bielli (2007)
			Mammalia	*Ovis canadensis*	Ungerfeld, Ramos & Bielli (2007)
	+ Social glue		Aves	*Cygnus atratus*	MacFarlane, Blomberg & Vasey (2010)
	Practice		Aves	*Ptilonorhynchus violaceus*	MacFarlane, Blomberg & Vasey (2010)
	Displaced behaviour		Mammalia	*Capra hircus*	Ungerfeld et al. (2014)
	Mistaken identity		Insecta	*Aphidius ervi*	Scharf & Martin (2013)
			Insecta	*Byrsotria fumigata*	Scharf & Martin (2013)
			Insecta	*Cotesia rubecula*	Scharf & Martin (2013)
			Insecta	*Euphydryas editha*	Scharf & Martin (2013)
			Insecta	*Eupoecilia ambiguella*	Scharf & Martin (2013)
		+ Displaced behaviour	Actinopterygii	*Oreochromis mossambicus*	Oliveira & Almada (1998); Amorim, Fonseca & Almada (2003)
		Thermoregulation	Reptilia	*Thamnophis sirtalis parietalis*	Bailey & Zuk (2009); Shine et al. (2000)

**Note:**

Species for which the competition hypothesis was rejected are not included in this table (i.e., *Gryllus veletis* and *Teleogryllus oceanicus* mentioned in Table 2).

### Bystander-related behaviours

We found evidence of bystander-related behaviour in 19 out of the 71 species analysed (26.8%) (see Table 4 and Table S2): nine species out of 20 for which the intrasexual competition hypothesis was suggested and 10 species out of 51 for which other hypotheses were suggested. For species that have been linked to intrasexual competition, we detected evidence of eavesdropping in nine, exploitation in five and audience effect in five. For the 51 species that have been linked to another hypothesis, we found evidence of eavesdropping for six, exploitation for four and audience effect for five.

**Table 4:** Species per MMCD explanatory hypothesis and bystander-related behaviours (BRB).

MMCD hypothesis	Species names	Bystander-related behaviours (BRB)		
		Eavesdropping	Exploitation	Audience effect
Competition	*Aphidius ervi*	−	−	−
	*Byrsotria fumigata*	−	−	−
	*Cephalonomia tarsalis*	−	−	−
	*Cotesia rubecula*	−	−	−
	*Cygnus atratus*	−	−	−
	*Euphydryas editha*	−	−	−
	*Eupoecilia ambiguella*	−	−	−
	*Melanerpes formicivorus*	−	−	−
	*Melopsittacus undulatus*	−	−	−
	*Poephila acuticauda*	−	−	−
	*Teleogryllus occipitalis*	−	−	−
	*Rupicola rupicola*	*+*	−	−
	*Oreochromis mossambicus*	*+*	*+*	−
	*Ovis aries*	*+*	*+*	−
	*Ptilonorhynchus violaceus*	*+*	*+*	−
	*Capra hircus*	*+*	−	*+*
	*Taeniopygia guttata*	*+*	−	*+*
	*Gnatocerus cornutus*	*+*	−	*+*
	*Ovis canadensis*	*+*	*+*	*+*
	*Thamnophis sirtalis parietalis*	*+*	*+*	*+*
	**Total**	**9**	**5**	**5**
Other	*Aegus chelifer*	−	−	−
	*Allomyrina dichotoma*	−	−	−
	*Bemisia tabaci*	−	−	−
	*Cerotainia albipilosa*	−	−	−
	*Choristoneura fumifera*	−	−	−
	*Columba livia (f. urbana)*	−	−	−
	*Corynorhinus rafinesquii*	−	−	−
	*Dacus cucurbitae*	−	−	−
	*Desmodus rotundus*	−	−	−
	*Drosophila affinis*	−	−	−
	*Drosophila ananassae*	−	−	−
	*Drosophila erecta*	−	−	−
	*Drosophila birchii*	−	−	−
	*Drosophila heteroneura*	−	−	−
	*Drosophila montana*	−	−	−
	*Drosophila persimilis*	−	−	−
	*Drosophila silvestris*	−	−	−
	*Eptesicus serotinus*	−	−	−
	*Eurycotis floridana*	−	−	−
	*Glossina morsitans*	−	−	−
	*Grapholitha molesta*	−	−	−
	*Hermetia illucens*	−	−	−
	*Lariophagus distinguendus*	−	−	−
	*Magicicada cassini*	−	−	−
	*Magicicada septendecim*	−	−	−
	*Menura novaehollandiae*	−	−	−
	*Musca domestica*	−	−	−
	*Myotis myotis*	−	−	−
	*Nyctalus noctula*	−	−	−
	*Oedothorax fuscus*	−	−	−
	*Oedothorax gibbosus*	−	−	−
	*Periplaneta americana*	−	−	−
	*Periplaneta brunnea*	−	−	−
	*Phytoecia rufiventris*	−	−	−
	*Pieris rapae crucivora*	−	−	−
	*Prochyliza xanthostoma*	−	−	−
	*Protophormia terraenovae*	−	−	−
	*Psyttalia concolor*	−	−	−
	*Pteropus giganteus*	−	−	−
	*Testudo hermanni*	−	−	−
	*Thyanta pallidovirens*	−	−	−
	*Bactrocera oleae*	*+*	−	−
	*Gryllus bimaculatus*	*+*	−	−
	*Hylobittacus apicalis*	−	*+*	−
	*Chrysoperla lucasina*	−	−	+
	*Gryllus veletis*	−	−	+
	*Euscepes postfasciatus*	−	−	+
	*Ceratitis capitata*	*+*	*+*	−
	*Teleogryllus oceanicus*	*+*	*+*	−
	*Megacopta punctatissima*	*+*	−	*+*
	*Drosophila melanogaster*	*+*	*+*	*+*
** **	**Total**	**6**	**4**	**5**

**Note:**

Species for which the intrasexual competition and other explanatory hypotheses have been suggested to explain male-male courtship display (MMCD), and evidence of each bystander-related behaviours (BRB): eavesdropping, exploitation, and audience effect. Evidence or no evidence found per each BRB is represented as “+” or as “−”, respectively.

**Figure 4:**
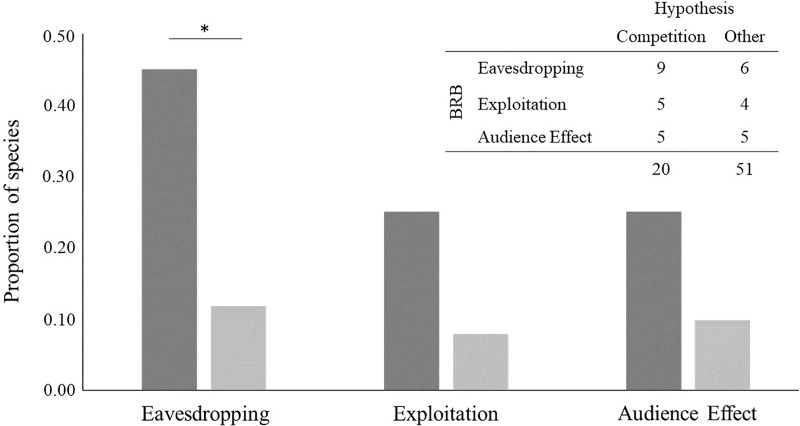
Bystander-related behaviours (BRB) per hypothesis suggested to explain the evolution of male-male courtship display (MMCD). BRBs are (from left to right in the x axis): eavesdropping and exploitation of male-female courtship interactions by bystander males, and audience effect by the displayer male towards the bystander. The species considered are the ones found in the first systematic review, excluding those for which no hypothesis of MMCD was suggested (*n* = 71). The y-axis is the proportion of species, calculated based on the frequency of species represented in the table at the top right. Dark grey bars represent the proportion of species for which the intrasexual competition hypothesis was suggested, and light grey bars the proportion of species for which other hypotheses were suggested. *p < 0.01.

Evidence of eavesdropping was significantly more frequent in species for which the intrasexual competition hypothesis has been suggested (Fisher’s exact test: *p* = 0.00383, *n* = 71; Fig. 4). It was 3.8 times more likely to find evidence of this bystander-related behaviour for species that male-male courtship display has been related to intrasexual competition than to any other hypothesis. The result was similar after removing species for which more than one hypothesis, in addition to competition, has been suggested (*p* = 0.01211, *n* = 61). By contrast, evidence for other bystander-related behaviour was independent of the suggested male-male courtship display hypotheses, either when considering all species or when excluding species for which the intrasexual competition hypothesis was suggested together with other hypotheses (Fisher’s exact tests for exploitation: *p* = 0.1051, *n* = 71 and *p* = 0.2526, *n* = 61; Fisher’s exact tests for audience effect: *p* = 0.1314, *n* = 71 and *p* = 0.1154, *n* = 61; Fig. 4). Concerning the analysis with all bystander-related behaviour combined, we found similar results to the analysis with only eavesdropping: all bystander-related behaviours combined were significantly more frequent in species for which the intrasexual competition hypothesis has been suggested (Fisher’s exact test for combined bystander-related behaviour: *p* = 0.01035, *n* = 71), being 7.5 times more likely to find evidence of the three combined bystander-related behaviours than evidence of none for species related with the intrasexual competition. This result was similar even after removing species for which the intrasexual competition hypothesis has been suggested together with another explanation (Fisher’s exact test for combined bystander-related behaviours: *p* = 0.04924, *n* = 71). Taken together, these results support that male-male courtship display can result from a competition pressure likely driven by the eavesdropping effect.

## Discussion

This review supports an evolutionary hypothesis to explain the origin of male-male courtship display—a same-sex sexual behaviour that has long puzzled researchers. We propose bridging the knowledge and conceptual frameworks of two research fields: sexual signals’ dual function (Berglund, Bisazza & Pilastro, 1996) and same-sex sexual behaviours (Bailey & Zuk, 2009). In doing so, we hypothesize that the male-male courtship display has evolved as a secondary function from the male-female courtship display. For this to happen, the evolutionary mechanism should have been intrasexual competition due to bystander male pressure during male-female courtship interactions. Using data from two systematic reviews, the results suggest that our hypothesis could be correct, therefore deserving the attention of future empirical studies.

The first systematic review focused on male-male courtship display studies. Studies describing male-male courtship display are divided between those that suggest and that do not suggest any hypothesis to explain this behaviour. The most frequent hypothesis is “mistaken identity”. This non-adaptive hypothesis is likely in taxa with incomplete mate recognition mechanisms—namely low sexual dimorphism—, or when there is interference from male-female sexual interactions (Bailey & Zuk, 2009; Balfour & Shuker, 2020). For example, male insects may mistake a male for a female due to the transmission of female sexual pheromones to males during previous mating (Scharf & Martin, 2013). Concerning adaptive explanations, the intrasexual competition hypothesis was the most frequently suggested: we found 14 studies describing 20 species. However, only four studies formally tested this hypothesis (Boutin et al., 2016; Lane et al., 2016; Kuriwada, 2017; Rayner & Bailey, 2019), from which only two found evidence to support it (Lane et al., 2016; Kuriwada, 2017). Therefore, our first review highlights the need to empirically test the intrasexual competition hypothesis in more species with male-male courtship display. The current empirical record is not enough to allow drawing safe conclusions about the role of intrasexual competition in this behaviour.

The second systematic review focused on studies about bystander-related behaviours. As predicted, eavesdropping was more prevalent in species for which the intrasexual competition hypothesis has been suggested. However, we failed to find a similar significant association between other bystander-related behaviours (exploitation and audience effect) and the intrasexual competition hypothesis. This may happen due to lack of data, as exploitation and audience effect are probably more frequent in nature than reported. In fact, while eavesdropping is overall easily observed and assumed by the mere presence of a bystander in a displayer male’s territory, evidence for exploitation or audience effect requires a specific examination of these behaviours. For example, we can assume that males eavesdrop on other males’ courtship displays in lekking species. Still, we can only know that a bystander exploits this information and that a displayer male modifies his behaviour accordingly with an empirical approach (Dabelsteen, 2005; Zuberbühler, 2008). It is also possible that exploitation and audience effect have only appeared at some stage during the evolution of male-male courtship display but have since disappeared. Because exploitation and audience effect benefit one individual but harm the other, these behaviours are less likely to be evolutionarily stable than eavesdropping (Bradbury & Vehrencamp, 2000). For example, in three-spined stickleback *Gasterosteus aculeatus* displayer males conceal their courtship displays from bystanders to avoid exploitation (Dzieweczynski & Rowland, 2004). However, despite this result for exploitation and audience effect independently, we still found a significant association of all three bystander-related behaviours combined with the intrasexual competition hypothesis, which is again consistent with a male-male courtship display competition-driven evolution. Therefore, our second review highlights the need to empirically test the effect of male bystanders on the evolution of male-male courtship display and, hence, on the evolution of courtship display dual function.

Despite using systematic review methodology, some studies may still have been missed in our analyses. For instance, male-male courtship display has been described for several poeciliid fish (Goldberg et al., 2019), but our systematic reviews only retrieved studies in guppies (*Poecilia reticulata*; Bailey & Zuk, 2009). This likely resulted from not including specific terms used for male courtship display in poeciliid fish, like “S-shaped body posture” or “sigmoid behaviour” (*e.g*., Farr, 1984; Ptacek, 1998). Thus, we would have had to include specific terms of male courtship display for all taxa to improve our review lists potentially. Nonetheless, we are confident that the most relevant studies were likely included as we searched generalist and inclusive terms used by researchers. However, to achieve a more comprehensive view of the evolution of male-male courtship display and same-sex sexual behaviours in general, we suggest that researchers include general terms, and not only species-specific terms, in the keywords and abstracts of their publications.

So, empirical studies on male-male courtship display are scarce, making it difficult to evaluate whether it evolved by intrasexual competition, and if yes, whether it evolved primarily with a competitive function or gained such a function secondarily. Our study can help advancing this research area, as it makes the 20 species for which the intrasexual competition hypothesis was suggested excellent candidates to formally test the intrasexual competition hypothesis where it has not yet been tested. The same species are also good models to study male courtship display dual function, especially the nine species for which there is also evidence of bystander-related behaviours.

The intrasexual competition hypothesis predicts that after a male-male courtship interaction the male that received the courtship should reduce the frequency or the success of his future mating interactions with females or abandon the displayer male’s territory (Yorzinski et al., 2017). If that is the case, this would be evidence of a loser effect resulting from the interaction with the displayer male. Contrastingly, the displayer male should show evidence of a winner effect by immediately or indirectly gaining higher mating success due to acquiring a higher hierarchical position and prior access to breeding resources (a territory or a female; Abbassi & Burley, 2012; Lane et al., 2016). On the other hand, if both displayer and bystander males increase their ability to mate, other functions for male-male courtship display must be considered—such as courtship practice or improved sexual discrimination (Abbassi & Burley, 2012; Harari, Brockmann & Landolt, 2000; respectively).

When studying male courtship display dual function, the prediction for male-male courtship display is similar: winner effect by the displayer and loser effect by the bystander. The goal, however, is to identify the primary function of the sexual signal, that is, whether it evolved first by inter or intrasexual competition. Hence, if male courtship display has a dual function, it must be displayed both in the presence of a female and of a rival male. However, it may be more frequently used in one context than in the other, which is what helps identify its primary function (Albrecht & Oring, 1995). Contrastingly, if the male courtship display has no dual function then: it must be displayed only in the presence of a female or of a rival male; or have no effect on attracting females or intimidating rivals (Pope, 2000; Delaney, Roberts & Uetz, 2007). We know that the male courtship display can be directed towards both females and males, and it seems to have been primarily favoured by intersexual selection, because it is more frequently directed to females than to males. However, whether it has an effective role in intimidating rivals is what remains to be empirically studied in a more significant number of species.

Complementary to empirical studies, we also suggest researchers conduct comparative analyses with the species list we provide to test correlated evolution along branches of a phylogeny between male courtship display and bystander-related behaviours, as well as for ancestral state reconstructions (Pagel, 1999). This type of method would allow testing the evolutionary transitions, as well as the order of evolutionary steps between male-female courtship interactions and male-male courtship display *via* bystander pressure suggested in Fig. 1. The male courtship display represents, indeed, an excellent candidate to study the dual function of sexual traits, given its commonness across the animal kingdom and the increasing records of male-male courtship display.

## Conclusion

Studying the adaptive significance of behavioural traits is fundamental to understanding how past selective forces have shaped species’ evolution (Tinbergen, 1963; Nesse, 2013). However, it can be challenging to distinguish a behaviour’s current utility if it has been more recently co-opted to another function (Bateson & Laland, 2013). This is the case for male-male courtship display, which may have evolved as a new behaviour or function of male-female courtship. Testing the dual function hypothesis in species with male-male courtship display merges the interests and conceptual approaches of these two research areas, and can lead to a more comprehensive understanding of male courtship display and same-sex sexual behaviour evolution.

## Supplemental Information

10.7717/peerj.14638/supp-1 Supplemental Information 1 Evidence of hypotheses suggested to explain male-male courtship display by species.

10.7717/peerj.14638/supp-2 Supplemental Information 2 Evidence of bystander-related behaviours (eavesdropping, exploitation, and audience effect) during male-female courtship display interactions for species with male-male courtship display.

10.7717/peerj.14638/supp-3 Supplemental Information 3 References for the systematic reviews.

10.7717/peerj.14638/supp-4 Supplemental Information 4 Script with statistical analyses and results.

10.7717/peerj.14638/supp-5 Supplemental Information 5 Data for the analyses of each behaviour independently. Data to test if each bystander-related behaviour (eavesdropping, exploitation and audience effect) differed according to the intrasexual competition hypothesis

10.7717/peerj.14638/supp-6 Supplemental Information 6 PRISMA checklist.

10.7717/peerj.14638/supp-7 Supplemental Information 7 Rationale and contribution of the systematic reviews.

## Additional Information and Declarations

### Data Availability

The following information was supplied regarding data availability: The raw data and code are available in the Supplemental Files.
