# Supplementary material for: The role of intrasexual competition on the evolution of male-male courtship display: a systematic review
Source: PeerJ. 2023 Feb 2;10:e14638. doi: 10.7717/peerj.14638 (PMC9899439; doi:10.7717/peerj.14638)
Supplement: Supplemental Information 2 [file peerj-11-14638-s002.docx]

| **Species** | **Common name** | **Hypothesis suggested** | **Eavesdropping** | **Exploitation** | **Audience effect** |
| --- | --- | --- | --- | --- | --- |
| *Aegus chelifer chelifer* | stag beetle | other |  |  |  |
| *Allomyrina dichotoma septentrionalis* | japanese horned beetle | other |  |  |  |
| *Aphidius ervi* | parasitoid wasp (of aphids) | competition |  |  |  |
| *Bactrocera oleae* | olive fruit fly | other | Benelli et al. (2014, 2016) |  |  |
| *Bemisia tabaci* | silverleaf whitefly | other |  |  |  |
| *Byrsotria fumigata* | cuban burrowing cockroach | competition |  |  |  |
| *Capra hircus* | bucks | competition | Price et al. (1991) |  | Lacuesta and Ungerfeld (2012) |
|  |  |  | Fritz et al. (2019) |  |  |
|  |  |  | Ungerfeld et al. (2019) |  |  |
| *Cephalonomia tarsalis* | parasitoid wasp (of beetles) | competition |  |  |  |
| *Ceratitis capitata* | mediterranean fruitfly or medfly | other | Papanastasiou et al. (2011) | Papanastasiou et al. (2011) |  |
|  |  |  | Hendrichs et al. (1994) | Shelly (2000) |  |
|  |  |  | Shelly (2000) |  |  |
|  |  |  | Benelli et al. (2014) |  |  |
|  |  |  | Gobbi et al. (2020) |  |  |
| *Cerotainia albipilosa* | robber fly | other |  |  |  |
| *Choristoneura fumifera* | eastern spruce budworm | other |  |  |  |
| *Chrysoperla lucasina* | green lacewing | other |  |  | Noh and Henry (2015) |
| *Columbia livia (f. urbana)* | (feral) pigeon | other |  |  |  |
| *Corynorhinus rafinesquii* | Rafinesque's big-eared bat | other |  |  |  |
| *Cotesia rubecula* | parasitoid wasp (of butterflies) | competition |  |  |  |
| *Cygnus atratus* | black swan | competition |  |  |  |
| *Dacus cucurbitae* | wild melon fly | other |  |  |  |
| *Desmodus rotundus* | (common) vampire bat | other |  |  |  |
| *Drosophila affinis* | (common) fruit fly | other |  |  |  |
| *Drosophila ananassae* | fruit fly spp. | other |  |  |  |
| *Drosophila erecta* | fruit fly spp. | other |  |  |  |
| *Drosophila birchii* | fruit fly spp. | other |  |  |  |
| *Drosophila heteroneura* | fruit fly | other |  |  |  |
| *Drosophila melanogaster* | (common) fruit fly | other | Maguire and Price (2015) | Ng et al. (2014) | Rouse and Bretman (2016) |
|  |  |  | Churchill et al. (2020) | Churchill et al. (2020) | Churchill et al. (2020) |
|  |  |  | Ishikawa et al. (2019) |  | Ishikawa et al. (2019) |
| *Drosophila montana* | fruit fly spp. | other |  |  |  |
| *Drosophila paulistorum* | leaf-mining tephritid fly, eventually fruit fly | other |  |  |  |
| *Drosophila silvestris* | european grape berry moth, eventually fruit fly | other |  |  |  |
| *Euscepes postfasciatus* | West Indian sweetpotato weevil | other |  |  | Kumano et al. (2010) |
| *Eptesicus serotinus* | serotine bat | other |  |  |  |
| *Euphydryas editha* | Edith's checkerspot | competition |  |  |  |
| *Eupoecilia ambiguella* | scorpion fly | competition |  |  |  |
| *Eurycotis floridana* | parasitoid wasp (of weevils) | other |  |  |  |
| *Glossina morsitans morsitans* | pharaoh cicada | other |  |  |  |
| *Gnatocerus cornutus* | honed beetle or broadhorned flour beetle | competition | Lane et al. (2015) |  |  |
| *Grapholitha molesta* | dwarf cicada | other |  |  |  |
| *Gryllus bimaculatus* | black cricket | other | Schoneich (2020) |  |  |
| *Gryllus veletis* | spring field cricket | other |  |  | Boutin et al. (2016) |
| *Hermetia illucens* | black soldier fly | other |  |  |  |
| *Hylobittacus apicalis* | plataspid stinkbug | other |  | Thornhill (1980) |  |
| *Lariophagus distinguendus* |  | other |  |  |  |
| *Magicicada cassini* | cassini/dwarf periodical cicada | other |  |  |  |
| *Magicicada septendecim* | decim periodical cicada / pharaoh cicada | other |  |  |  |
| *Megacopta punctatissima* | true bug spp. / (japanese common) plataspid stink bug | other | Himuro et al. (2006) |  | Himuro et al. (2006) |
| *Melanerpes formicivorus* | acorn woodpecker | competition |  |  |  |
| *Melopsittacus undulatus* | budgerigars | competition |  |  |  |
| *Menura novaehollandiae* | (superb) lyrebird | other |  |  |  |
| *Musca domestica* | house-fly | other |  |  |  |
| *Myotis myotis* | greater mouse-eared bat / large mouse-eared bat / mouse-eared bat / mouse-eared myotis | other |  |  |  |
| *Nyctalus noctula* | (common) noctule | other |  |  |  |
| *Oedothorax fuscus* | dwarf spiders spp. | other |  |  |  |
| *Oedothorax gibbosus* | dwarf spiders spp. | other |  |  |  |
| *Oreochromis mossambicus* | mozambique tilapia | competition | Wackermannova et al. (2017) | Nelson (1995) |  |
| *Ovis aries* | lamb | competition | Ungerfeld et al. (2007) | Price et al. (1991) |  |
|  |  |  | Price et al. (1998) |  |  |
| *Ovis canadensis* | ram | competition | Ungerfeld et al. (2019) | Ungerfeld and Lacuesta (2015) | Ungerfeld and Lacuesta (2015) |
|  |  |  |  |  | Lacuesta and Ungerfeld (2012) |
|  |  |  |  |  | Ungerfeld (2012) |
|  |  |  |  |  | Ungerfeld and González‐Pensado (2009) |
| *Periplaneta americana* | american cockroach | other |  |  |  |
| *Periplaneta brunnea* | brown cockroach | other |  |  |  |
| *Phytoecia rufiventris* |  | other |  |  |  |
| *Pieris rapae crucivora* | white cabbage butterfly | other |  |  |  |
| *Poephila acuticauda* | Edith's checkerspot | competition |  |  |  |
| *Prochyliza xanthostoma* | northern blowfly | other |  |  |  |
| *Protophormia terrae-novae* | parasitoid wasp (of fruit flies) | other |  |  |  |
| *Psyttalia concolor* |  | other |  |  |  |
| *Pteropus giganteus* | indian flying(-)fox | other |  |  |  |
| *Ptilonorhynchus violaceus* | satin bowerbird | competition | Reynolds et al. (2009) | Borgia and Mueller (1992) |  |
|  |  |  | Borgia and Mueller (1992) |  |  |
| *Rupicola rupicola* | guianan cock-of-the-rock | competition | Omena Junior (2009) |  |  |
|  |  |  | Trail (1990) |  |  |
| *Taeniopygia guttata* | zebra finch | competition | Lin et al. (2014) |  | Jesse and Riebel (2012) |
|  |  |  | Jesse and Riebel (2012) |  | Gleeson (2007) |
|  |  |  | Jarvis et al. (1998) |  | Vignal et al. (2004) |
|  |  |  | Rodríguez-Saltos (2017) |  | Jarvis et al. (1998) |
|  |  |  |  |  | Dunn and Zann (1997) |
|  |  |  |  |  | Martin and Burley (2021) |
| *Teleogryllus occipitalis* |  | competition |  |  |  |
| *Teleogryllus oceanicus* | australian (oceanic/pacific field) cricket | other | Lane et al. (2015) | Tinghitella and Zuk (2009) |  |
|  |  |  | Reichard and Anderson (2015) |  |  |
| *Testudo hermanni* | Hermann’s tortoise | other |  |  |  |
| *Thamnophis sirtalis parietalis* | (red-sided) garter snakes | competition | Shine et al. (2005a) | Shine et al. (2005a) | Shine et al. (2005a, b) |
|  |  |  |  |  | Friesen et al. (2017) |
| *Thyanta pallidovirens* | red-shouldered stinkbug | other |  |  |  |
