# Supplementary material for: The role of intrasexual competition on the evolution of male-male courtship display: a systematic review
Source: PeerJ. 2023 Feb 2;10:e14638. doi: 10.7717/peerj.14638 (PMC9899439; doi:10.7717/peerj.14638)
Supplement: Supplemental Information 3 [file peerj-11-14638-s003.docx]

## References S1. Systematic review of male-male courtship display interactions (SR1).

### References S1.1. List of identified references.

1. Abbassi, P., and Burley, N.T. (2012). Nice guys finish last: Same-sex sexual behavior and pairing success in male budgerigars. Behav. Ecol. 23, 775–782. doi: 10.1093/beheco/ars030
2. Adachi, H., and Soma, M. (2019). Vocalization can mediate male-male sexual interactions in Java sparrows. Anim. Biol. J, 69(1), 5–15. doi: 10.1163/15707563-00001051
3. Adkins-Regan, E. (2014). Male-male sexual behavior in Japanese quail: Being “on top” reduces mating and fertilization with females. Behavioural processes. 108, 71-79. doi: 10.1016/j.beproc.2014.09.027
4. Adriaens, P. R., and De Block, A. (2006). The evolution of a social construction: the case of male homosexuality. Perspectives in biology and medicine. 49(4), 570-585. doi: 10.1353/pbm.2006.0051
5. Agrawal, S., and Riffell, J.A. (2011). Behavioral neurobiology: the bitter life of male flies. Curr. Biol. 21, R470–R472. doi: 10.1016/j.cub.2011.05.024
6. Alanko, K., Santtila, P., Witting, K., Varjonen, M., Jern, P., Johansson, A., ... and Kenneth Sandnabba, N. (2009). Psychiatric symptoms and same-sex sexual attraction and behavior in light of childhood gender atypical behavior and parental relationships. Journal of Sex Research. 46(5), 494-504. doi: 10.1080/00224490902846487
7. Amorim, M.C.P., Fonseca, P.J., and Almada, V.C. (2003). Sound production during courtship and spawning of *Oreochromis mossambicus*: male-female and male-male interactions. J. Fish Biol. 62, 658–672. doi: 10.1046/j.0022-1112.2003.00054.x
8. Anaka, M., Anaka, M., Macdonald, C.D., Barkova, E., Simon, K., Rostom, R., Godoy, R. a., Haigh, A.J., Meinertzhagen, I. A., and Lloyd, V. (2008). The white Gene of Drosophila melanogaster encodes a protein with a role in courtship behavior. J. Neurogenet. 22, 243–276. doi: 10.1080/01677060802309629
9. Bailey, N.W., and French, N. (2012). Same-sex sexual behaviour and mistaken identity in male field crickets, *Teleogryllus oceanicus*. Anim. Behav. 84. doi: 10.1016/j.anbehav.2012.08.001
10. Bailey, N.W., and Zuk, M. (2009). Same-sex sexual behavior and evolution. Trends Ecol. Evol. 24, 439–446. doi: 10.1016/j.tree.2009.03.014
11. Bailey, N.W., Hoskins, J.L., Green, J., and Ritchie, M.G. (2013). Measuring same-sex sexual behaviour: the influence of the male social environment. Anim. Behav. 86, 91–100. doi: 10.1016/j.anbehav.2013.04.016
12. Barron, A. B., and Hare, B. (2020). Prosociality and a sociosexual hypothesis for the evolution of same-sex attraction
13. Benelli, G., and Canale, A. (2013). Male-male sexual behavior in the parasitic wasp *Psyttalia concolor*. J. Insect Sci. 13, 25. doi:
14. Benelli, G., Bonsignori, G., Stefanini, C., and Canale, A. (2012). Courtship and mating behaviour in the fruit fly parasitoid *Psyttalia concolor* (Szépligeti) (Hymenoptera: Braconidae): the role of wing fanning. J. Pest Sci. (2004). 85, 55–63. doi: 10.1007/s10340-011-0394-x
15. Benelli, G., Bonsignori, G., Stefanini, C., Raspi, A., and Canale, A. (2013). The production of female sex pheromone in *Bactrocera oleae* (Rossi) young males does not influence their mating chances. Entomol. Sci. 16, 47–53. doi: 10.1111/j.1479-8298.2012.00538.x
16. Benvenga, S. (2005). Central hormonal regulation and dimorphism of arousal. International journal of andrology. 28, 18-22. doi: 10.1111/j.1365-2605.2005.00583.x
17. Berger, D., You, T., Minano, M. R., Grieshop, K., Lind, M. I., Arnqvist, G., and Maklakov, A. A. (2016). Sexually antagonistic selection on genetic variation underlying both male and female same-sex sexual behavior. BMC Evol Biol. 16(88), 1–11. [doi: 10.1186/s12862-016-0658-4](https://doi.org/10.1186/s12862-016-0658-4)
18. Bierbach, D., Jung, C. T., Hornung, S., Streit, B., and Plath, M. (2013). Homosexual behaviour increases male attractiveness to females. Biology Letters. 9(1), 20121038. doi: 10.1098/rsbl.2012.1038
19. Bishop, M. D., Fish, J. N., Hammack, P. L., and Russell, S. T. (2020). Sexual identity development milestones in three generations of sexual minority people: a national probability sample. Dev. Psychol. 56(11), 2177–2193. [doi: 10.1037/dev0001105.](https://doi.org/10.1037/dev0001105.Sexual)
20. Blohowiak, C. C., Zelenka, D. J., and Siegel, P. B. (1985). Ontogeny of aggressive-sexual behavior between males in lines of Japanese quail (*Coturnix coturnix japonica*) selected for male mating frequency. Journal of Comparative Psychology. 99(1), 30. doi: 10.1037/0735-7036.99.1.30
21. Blumberg, S. J., Cynamon, M. L., Osborn, L., and Olson, L. (2003). The impact of touch‐tone data entry on reports of HIV and STD risk behaviors in telephone interviews.
22. Bonnet, X., Golubović, A., Arsovski, D., Dević, S., Ballouard, J. M., Sterijovski, B., Ajtić, R., Barbraud, C., and Tomović, L. (2016). A prison effect in a wild population: a scarcity of females induces homosexual behaviors in males. Behav. Ecol. 27(4), 1206–1215. doi: 10.1093/beheco/arw023
23. Boutin, S.R.T., Harrison, S.J., Fitzsimmons, L.P., McAuley, E.M., and Bertram, S.M. (2016). Same-sex sexual behaviour in crickets: understanding the paradox. Anim. Behav. 114, 101–110. doi: 10.1016/j.anbehav.2016.01.022
24. Boyer, C. Reyn, and Galupo, M.P. (2015). ‘Prove it!’ Same-sex performativity among sexual minority women and men. Psychology and Sexuality. 6.4, 357-368. doi: 10.1080/19419899.2015.1021372 doi: 10.1080/19419899.2015.1021372
25. Brewer, M. (2018). Good Ol’ country boys playin’ on the farm: online articulations of rural masculinity by men who have sex with men. Sex Cult. 22(2), 355–379. [doi: 10.1007/s12119-017-9470-6](https://doi.org/10.1007/s12119-017-9470-6)
26. Brooker, J. S., Webb, C. E., and Clay, Z. (2020). Fellatio among male sanctuary-living chimpanzees during a period of social tension. Behaviour. 158(1), 77–87. doi: 10.1163/1568539X-bja10053
27. Burgevin, L., Friberg, U., and Maklakov, A. A. (2013). Intersexual correlation for same-sex sexual behaviour in an insect. Animal Behaviour. 85(4), 759-762. doi: 10.1016/j.anbehav.2013.01.017
28. Button, D. M., and Worthen, M. G. (2014). General strain theory for LGBQ and SSB youth: The importance of intersectionality in the future of feminist criminology. Feminist criminology. 9(4), 270-297. doi: 10.1177/1557085114525988
29. Caballero-Mendieta, N., and Cordero, C. (2012). Enigmatic liaisons in Lepidoptera: A review of same-sex courtship and copulation in butterflies and moths. Journal of Insect Science. 12(1), 138.
30. Caballero-Mendieta, N., and Cordero, C. (2012). Enigmatic liaisons in Lepidoptera: A review of same-sex courtship and copulation in butterflies and moths. Journal of Insect Science, 12(1), 138.
31. Cardoso, F. L. (2009). Similar faces of same-sex sexual behavior: a comparative ethnographical study in Brazil, Turkey, and Thailand. J Homosex. 56(4), 457-484. doi: 10.1080/00918360902816866
32. Certel, S.J., Leung, A., Lin, C.Y., Perez, P., Chiang, A.S., and Kravitz, E.A. (2010). Octopamine neuromodulatory effects on a social behavior decision-making network in Drosophila males. PLoS One 5. doi: 10.1371/journal.pone.0013248
33. Chae, D., Krieger, N., Bennett, G., Lindsey, J., and Stoddard, A. E. Barbeau. 2010.“Implications of Discrimination Based on Sexuality, Gender, and Race/Ethnicity for Psychological Distress Among Working-Class Sexual Minorities: The United for Health Study, 2003–2004.” Work and Health. 40(4), 589-608. doi: 10.2190/HS.40.4.b
34. Chan, Y.-B., and Kravitz, E.A. (2007). Specific subgroups of Fru(M) neurons control sexually dimorphic patterns of aggression in Drosophila melanogaster. Proc. Natl. Acad. Sci. U. S. A. 104, 19577–19582. doi: 10.1073/pnas.0709803104
35. Chen, B., Liu, H., Ren, J., and Guo, A. (2012). Mutation of Drosophila dopamine receptor DopR leads to male-male courtship behavior. Biochem. Biophys. Res. Commun. 423, 557–563. doi: 10.1016/j.bbrc.2012.06.003
36. Chen, S. L., Chen, Y. H., Wang, C. C., Yu, Y. W., Tsai, Y. C., Hsu, H. W., Wu, C. L., Wang, P. Y., Chen, L. C., Lan, T. H., and Fu, T. F. (2017). Active and passive sexual roles that arise in Drosophila male-male courtship are modulated by dopamine levels in PPL2ab neurons. Sci. Rep.. 7(October 2016), 1–10. [doi: 10.1038/srep44595](https://doi.org/10.1038/srep44595)
37. Chertemps, T., François, A., Durand, N., Rosell, G., Dekker, T., Lucas, P., and Maïbèche-Coisne, M. (2012). A carboxylesterase, Esterase-6, modulates sensory physiological and behavioral response dynamics to pheromone in Drosophila. BMC Biol. 10, 56. doi: 10.1186/1741-7007-10-56
38. Chevalier-Skolnikoff, S. (1974). Male-female, female-female, and male-male sexual behavior in the stumptail monkey, with special attention to the female orgasm. Arch. Sex. 3(2), 95-116. doi: 10.1007/BF01540994
39. Ciani, A. C., and Battaglia, U. (2014). Implicit measurements of sexual preference in self‐declared heterosexual men: A pilot study on the rate of androphilia in Italy. The journal of sexual medicine. 11(9), 2207-2217. doi: 10.1111/jsm.12565
40. Ciani, A. S. C. (2021). The genetic inactivation of the vomero-nasal organ in primates allows the evolution of same-sex sexual behavior but does not explain homosexual orientation in humans. Archives of Sexual Behavior. 50(6), 2277–2281. [doi: 10.1007/s10508-020-01708-9](https://doi.org/10.1007/s10508-020-01708-9)
41. Clancy, L. M., Cooper, A. L., Griffith, G. W., and Santer, R. D. (2017). Increased male-male mounting behaviour in desert locusts during infection with an entomopathogenic fungus. Sci. Rep. 7(1), 1–7. doi: 10.1038/s41598-017-05800-4
42. Clark, J. L., Caceres, C. F., Lescano, A. G., Konda, K. A., Leon, S. R., Jones, F. R., *et al.* (2007). Prevalence of same-sex sexual behavior and associated characteristics among low-income urban males in Peru. PloS one. 2(8), e778. doi: 10.1371/journal.pone.0000778
43. Clive, J., Flintham, E., and Savolainen, V. (2020). Understanding same-sex sexual behaviour requires thorough testing rather than reinvention of theory. Nat. Ecol. Evol. 4(6), 784–785. doi: 10.1038/s41559-020-1189-3
44. Closson, E. F., Sivasubramanian, M., Mayer, K. H., Srivastava, A., Safren, S. A., Anand, V. R., *et al.* (2014). The other side of the bridge: exploring the sexual relationships of men who have sex with men and their female partners in Mumbai, India. Culture, health and sexuality. 16(7), 780-791. doi: 10.1080/13691058.2014.911960
45. Crossley, S.A., Bennet-Clark, H.C., and Evert, H.T. (1995). Courtship song components affect male and female Drosophila differently. Anim. Behav. 50, 827–839. doi: 10.1016/0003-3472(95)80142-1
46. Dai, H., Chen, Y., Chen, S., Mao, Q., Kennedy, D., Landback, P., Eyre-Walker, A., Du, W., and Long, M. (2008). The evolution of courtship behaviours through the origination of a new gene in Drosophila. Proc. Natl. Acad. Sci. U. S. A. 105, 7478–7483. doi: 10.1073/pnas.0800693105
47. Dalton, J.E., Lebo, M.S., Sanders, L.E., Sun, F., and Arbeitman, M.N. (2009). Ecdysone receptor acts in fruitless- expressing neurons to mediate Drosophila courtship behaviors. Curr. Biol. 19, 1447–1452. doi: 10.1016/j.cub.2009.06.063
48. de Almeida Neto, C., McFarland, W., Murphy, E. L., Chen, S., Nogueira, F. A. H., Mendrone Jr, A., *et al.* (2007). Risk factors for human immunodeficiency virus infection among blood donors in Sao Paulo, Brazil, and their relevance to current donor deferral criteria. Transfusion. 47(4), 608-614. doi: 10.1111/j.1537-2995.2007.01161.x
49. Deshpande, S. A., Meiselman, M., Hice, R. H., Arensburger, P., Rivera-Perez, C., Kim, D. H., Croft, R. L., Noriega, F. G., and Adams, M. E. (2019). Ecdysis triggering hormone receptors regulate male courtship behavior via antennal lobe interneurons in Drosophila. Gen. Comp. Endocrinol. 278(May 2018), 79–88. doi: 10.1016/j.ygcen.2018.12.003
50. Dickins, T. E., and Rahman, Q. (2020). Ancestral primacy of same-sex sexual behaviour does not explain its stable prevalence in modern populations. Nat. Ecol. Evol. 4(6), 782–783. [doi: 10.1038/s41559-020-1187-5](https://doi.org/10.1038/s41559-020-1187-5)
51. Dube, E. M. (2000). The role of sexual behavior in the identification process of gay and bisexual males. Journal of Sex Research. 37(2), 123-132.
52. Dukas, R. (2010). Causes and consequences of male-male courtship in fruit flies. Anim. Behav. 80, 913–919. doi: 10.1016/j.anbehav.2010.08.017
53. Dunkle, K. L., Jewkes, R. K., Murdock, D. W., Sikweyiya, Y., and Morrell, R. (2013). Prevalence of consensual male–male sex and sexual violence, and associations with HIV in South Africa: A population-based cross-sectional study. PLoS medicine. 10(6), e1001472. doi: 10.1371/journal.pmed.1001472
54. DuRant, R. H., Krowchuk, D. P., and Sinal, S. H. (1998). Victimization, use of violence, and drug use at school among male adolescents who engage in same-sex sexual behavior. The Journal of pediatrics. 133(1), 113-118.
55. Eddison, M. (2021). A genetic screen for Drosophila social isolation mutants and analysis of sex pistol. Sci. Rep. 11(1). [doi: 10.1038/s41598-021-96871-x](https://doi.org/10.1038/s41598-021-96871-x)
56. Ejima, A. (2015). Pleiotropic actions of the male pheromone cis-vaccenyl acetate in Drosophila melanogaster. J. Comp. Physiol. A Neuroethol. Sensory, Neural, Behav. Physiol. 201, 927–932. doi: 10.1007/s00359-015-1020-9
57. Eliason, M. J. (1993). AIDS-related stigma and homophobia: implications for nursing education. Nurse Educator. 18(6), 27-30. doi: 10.1097/00006223-199311000-00016
58. Elie, J.E., Mathevon, N., and Vignal, C. (2011). Same-sex pair-bonds are equivalent to male-female bonds in a life-long socially monogamous songbird. Behav. Ecol. Sociobiol. 65, 2197–2208. doi: 10.1007/s00265-011-1228-9
59. Engel, K. C., Männer, L., Ayasse, M., and Steiger, S. (2015). Acceptance threshold theory can explain occurrence of homosexual behaviour. Biology letters. 11(1), 20140603. doi: 10.1098/rsbl.2014.0603
60. Eskin, M., Kaynak-Demir, H., and Demir, S. (2005). Same-sex sexual orientation, childhood sexual abuse, and suicidal behavior in university students in Turkey. Arch. Sex. 34(2), 185-195. doi: 10.1007/s10508-005-1796-8
61. Faulkner, A. H., and Cranston, K. (1998). Correlates of same-sex sexual behavior in a random sample of Massachusetts high school students. American journal of public health. 88(2), 262-266. doi: 10.2105/AJPH.88.2.262
62. Flintham, E. O., Yoshida, T., Smith, S., Pavlou, H. J., Goodwin, S. F., Carazo, P., and Wigby, S. (2018). Interactions between the sexual identity of the nervous system and the social environment mediate lifespan in *Drosophila melanogaster*. Proc. R. Soc. B: Biol. Sci. 285(1892). [doi: 10.1098/rspb.2018.1450](https://doi.org/10.1098/rspb.2018.1450)
63. Frisell, T., Lichtenstein, P., Rahman, Q., and Långström, N. (2010). Psychiatric morbidity associated with same-sex sexual behaviour: influence of minority stress and familial factors. Psychological medicine. 40(2), 315-324. doi: 10.1017/S0033291709005996
64. Gaines, P., Tompkins, L., Woodard, C.T., and Carlson, J.R. (2000). quick-to-court, a Drosophila mutant with elevated levels of sexual behavior, is defective in a predicted coiled-coil protein. Genetics. 154, 1627–1637.
65. Galea, J. T., Kinsler, J. J., Galan, D. B., Calvo, G., Sánchez, H., Leon, S. R., et al. (2015). Factors associated with visible anogenital warts among HIV-uninfected Peruvian men who have sex with men and transwomen: a cross-sectional study. Sexually transmitted diseases. 42(4), 202-207. doi: 10.1097/OLQ.0000000000000253
66. Ganna, A., Verweij, K. J. H., Nivard, M. G., Maier, R., Busch, A. S., Abdellaoui, A., Guo, S., Fah, J., Lichtenstein, P., Auton, A., Harris, K. M., Gary, W., Martin, E. R., Sanders, A. R., Perry, J. R. B., Benjamin, M., and Zietsch, B. P. (2019). Large-scale GWAS reveals insights into the genetic architecture of same-sex sexual behavior. Science. 365(6456), 1–22. doi: 10.1126/science.aat7693
67. Ganna, A., Verweij, K. J. H., Nivard, M. G., Maier, R., Wedow, R., Busch, A. S., Abdellaoui, A., Guo, S., Sathirapongsasuti, J. F., Team, M. R., Lichtenstein, P., Lundström, S., Långström, N., Auton, A., Kathleen Mullan, H., Beecham, G. W., Martin, E. R., Sanders, A. R., Perry, J. R. B., et al. (2021). Comment on “Large-scale GWAS reveals insights into the genetic architecture of same-sex sexual behavior.” Science. 371(6536), eaba5693. [doi: 10.1126/science.aba5693](https://doi.org/10.1126/science.aba5693)
68. Ganter, G.K., Panaitiu, A.E., Desilets, J.B., Davis-Heim, J.A., Fisher, E.A., Tan, L.C.H., Heinrich, R., Buchanan, E.B., Brooks, K.M., Kenney, M.T., et al. (2011). Drosophila male courtship behavior is modulated by ecdysteroids. J. Insect Physiol. 57, 1179–1184. doi: 10.1016/j.jinsphys.2011.05.007
69. Ganter, G.K., Walton, K.L., Merriman, J.O., Salmon, M. V., Brooks, K.M., Maddula, S., and Kravitz, E.A. (2007). Increased male-male courtship in ecdysone receptor deficient adult flies. Behav. Genet. 37, 507–512. doi: 10.1007/s10519-006-9140-1
70. Genovart, M., Pradel, R., and Oro, D. (2012). Exploiting uncertain ecological fieldwork data with multi‐event capture–recapture modelling: an example with bird sex assignment. Journal of animal ecology. 81(5), 970-977. doi: 10.1111/j.1365-2656.2012.01991.x
71. Giasson, L. O., and Haddad, C. F. (2006). Social interactions in *Hypsiboas albomarginatus* (Anura: Hylidae) and the significance of acoustic and visual signals. Journal of Herpetology. 40(2), 171-180. doi: 10.1670/205-05A.1
72. Giunti, G., Campolo, O., Laudani, F., and Palmeri, V. (2018). Male courtship behaviour and potential for female mate choice in the black soldier fly *Hermetia illucens* L. (Diptera: Stratiomyidae). Entomol. Gen. 38(1), 29–46. [doi: 10.1127/entomologia/2018/0657](https://doi.org/10.1127/entomologia/2018/0657)
73. Glick, S. N., and Golden, M. R. (2010). Persistence of racial differences in attitudes toward homosexuality in the United States. Journal of acquired immune deficiency syndromes (1999). 55(4), 516. doi: 10.1097/QAI.0b013e3181f275e0
74. Goisauf, M., Akyüz, K., and Martin, G. M. (2020). Moving back to the future of big data-driven research: reflecting on the social in genomics. Humanit. 7(1), 1–9. [doi: 10.1057/s41599-020-00544-5](https://doi.org/10.1057/s41599-020-00544-5)
75. Guevara-Fiore, P., Stapley, J., and Watt, P. J. (2010). Mating effort and female receptivity: how do male guppies decide when to invest in sex? Behavioral ecology and sociobiology. 64(10), 1665-1672. doi: 10.1007/s00265-010-0980-6
76. Gupta, T., Morgan, H. R., Andrews, J. C., Brewer, E. R., and Certel, S. J. (2017). Methyl-CpG binding domain proteins inhibit interspecies courtship and promote aggression in Drosophila. Sci. Rep. 7(1), 1–12. [doi: 10.1038/s41598-017-05844-6](https://doi.org/10.1038/s41598-017-05844-6)
77. Hamer, D., Mustanski, B., Sell, R., Sanders, S. A., and Garcia, J. R. (2021). Comment on “Large-scale GWAS reveals insights into the genetic architecture of same-sex sexual behavior.” Science. 371(6536), eaba2941. doi: 10.1126/science.aba2941
78. Han, C. S., and Brooks, R. C. (2015). Same-sex sexual behaviour as a by-product of reproductive strategy under male–male scramble competition. Animal behaviour. 108, 193-197. doi: 10.1016/j.anbehav.2015.07.035
79. Han, C. S., and Brooks, R. C. (2015). The interaction between genotype and juvenile and adult density environment in shaping multidimensional reaction norms of behaviour. Functional Ecology. 29(1), 78-87. doi: 10.1111/1365-2435.12299
80. Han, C. S., Santostefano, F., and Dingemanse, N. J. (2016). Do social partners affect same-sex sexual behaviour in male water striders? Anim. Behav. 116, 53–59. doi: 10.1016/j.anbehav.2016.03.033
81. Herbenick, D., Reece, M., Schick, V., Sanders, S. A., Dodge, B., and Fortenberry, J. D. (2010). An event‐level analysis of the sexual characteristics and composition among adults ages 18 to 59: Results from a national probability sample in the United States. The Journal of Sexual Medicine. 7, 346-361. doi: 10.1111/j.1743-6109.2010.02012.x
82. Herek, G. M. (1999). AIDS and stigma. American behavioral scientist. 42(7), 1106-1116. doi: 10.1177/0002764299042007006
83. Hing, A.L., and Carlson, J.R. (1996). Male-male courtship behavior induced by ectopic expression of the Drosophila white gene: role of sensory function and age. J Neurobiol 30, 454–464.
84. Holloway, I. W., Padilla, M. B., Willner, L., and Guilamo-Ramos, V. (2015). Effects of minority stress processes on the mental health of Latino men who have sex with men and women: A qualitative study. Arch. Sex. 44(7), 2087-2097. doi: 10.1007/s10508-014-0424-x
85. Hoskins, J.L., Ritchie, M.G., and Bailey, N.W. (2015). A test of genetic models for the evolutionary maintenance of same-sex sexual behaviour. Proc. R. Soc. B Biol. Sci. 282, 20150429. doi: 10.1098/rspb.2015.0429
86. Hoving, H. J. T., Fernández-Álvarez, F., Portner, E. J., and Gilly, W. F. (2019). Same-sex sexual behaviour in an oceanic ommastrephid squid, *Dosidicus gigas* (Humboldt squid). Mar. Biol.. 166(3), 1–7. [doi: 10.1007/s00227-019-3476-6](https://doi.org/10.1007/s00227-019-3476-6)
87. Hoving, H. J., Bush, S. L., and Robison, B. H. A shot in the dark: same-sex sexual behaviour in a deep-sea. doi: 10.1098/rsbl.2011.0680
88. Isa, M., Kumano, N., and Tatsuta, H. (2019). When a male perceives a female: The effect of waxy components on the body surface on decision-making in the invasive pest weevil. R. Soc. Open Sci. 6(2). [doi: 10.1098/rsos.181542](https://doi.org/10.1098/rsos.181542)
89. James, W. H. (2005). Biological and psychosocial determinants of male and female human sexual orientation. Journal of Biosocial Science. 37(5), 555. doi: 10.1017/S0021932004007059
90. Jankowiak, Ł., Tryjanowski, P., Hetmański, T., and Skórka, P. (2018). Experimentally evoked same-sex sexual behaviour in pigeons: Better to be in a female-female pair than alone. Sci. Rep. 8(1), 1–7. [doi: 10.1038/s41598-018-20128-3](https://doi.org/10.1038/s41598-018-20128-3)
91. Jeffery, A. J., Shackelford, T. K., Zeigler-Hill, V., Vonk, J., and Mcdonald, M. (2019). The evolution of human female sexual orientation introduction to human sexual orientation. 71–86. [doi: 10.1007/s40806-018-0168-2](https://doi.org/10.1007/s40806-018-0168-2)
92. Kamath, A., McDonough, C. E., Monk, J. D., Lambert, M. R., and Giglio, E. (2020). A. Kamath et al. reply. Nat. Ecol. Evol. 4(6), 786–787. [doi: 10.1038/s41559-020-1188-](https://doi.org/10.1038/s41559-020-1188-)4
93. Keller, M., and Chamero, P. (2021). Is the loss of Trpc2 gene function a real working hypothesis for the emergence of same-sex sexual behavior in Old World Primates? Arch. Sex. 50(6), 2293–2297. [doi: 10.1007/s10508-020-01664-4](https://doi.org/10.1007/s10508-020-01664-4)
94. Kim, J. I., Lee, J. W., Lee, Y. A., Lee, D. H., Han, N. S., Choi, Y. K., *et al.* (2013). Sexual activity counteracts the suppressive effects of chronic stress on adult hippocampal neurogenesis and recognition memory. Brain research. 1538, 26-40. doi: 10.1016/j.brainres.2013.09.007
95. King, M., Green, J., Osborn, D. P., Arkell, J., Hetherton, J., and Pereira, E. (2005). Family size in white gay and heterosexual men. Arch. Sex, 34(1), 117-122. doi: 10.1007/s10508-008-9386-1
96. Kitamoto, T. (2002). Conditional disruption of synaptic transmission induces male-male courtship behavior in Drosophila. Proc. Natl. Acad. Sci. U. S. A. 99, 13232–13237. doi: 10.1073/pnas.202489099
97. Krstic, D., Boll, W., and Noll, M. (2013). Influence of the White locus on the courtship behavior of Drosophila males. PLoS One 8, e77904. doi: 10.1371/journal.pone.0077904
98. Kuhle, B. X., and Radtke, S. (2013). Born both ways: The alloparenting hypothesis for sexual fluidity in women. Evolutionary Psychology, 11(2), 147470491301100202.
99. Kuperberg, A., and Walker, A. M. (2018). Heterosexual college students who hookup with same-sex partners. Arch. Sex. 47(5), 1387–1403. [doi: 10.1007/s10508-018-1194-7](https://doi.org/10.1007/s10508-018-1194-7)
100. Kuriwada, T. (2017). Male–male courtship behaviour, not relatedness, affects the intensity of contest competition in the field cricket. Anim. Behav. 126, 217–220. [doi: 10.1016/j.anbehav.2017.02.009](https://doi.org/10.1016/j.anbehav.2017.02.009)
101. Lacaille, F., Hiroi, M., Twele, R., Inoshita, T., Umemoto, D., Manière, G., Marion-Poll, F., Ozaki, M., Francke, W., Cobb, M., et al. (2007). An inhibitory sex pheromone tastes bitter for Drosophila males. PLoS One. 2, 1–7. doi: 10.1371/journal.pone.0000661
102. Lane, S.M., Haughan, A.E., Evans, D., Tregenza, T., and House, C.M. (2016). Same-sex sexual behaviour as a dominance display. Anim. Behav. 114, 113–118. doi: 10.1016/j.anbehav.2016.01.005
103. Leca, J. B., Gunst, N., and Vasey, P. L. (2014). Male homosexual behavior in a free-ranging all-male group of Japanese macaques at Minoo, Japan. Arch. Sex. 43(5), 853-861. doi: 10.1007/s10508-014-0310-6
104. Lefevor, G. T., Sorrell, S. A., Kappers, G., Plunk, A., Schow, R. L., Rosik, C. H., and Beckstead, A. L. (2020). Same-Sex Attracted, Not LGBQ: The associations of sexual identity labeling on religiousness, sexuality, and health among mormons. J Homosex. 67(7), 940–964. doi: 10.1080/00918369.2018.1564006
105. Legate, N., and Rogge, R. D. (2019). Identifying Basic Classes of Sexual Orientation with Latent Profile Analysis: Developing the Multivariate Sexual Orientation Classification System. Arch. Sex, 48(5), 1403–1422. doi: 10.1007/s10508-018-1336-y
106. Lerch, B. A., and Servedio, M. R. (2021). Same-sex sexual behaviour and selection for indiscriminate mating. Nat. Ecol. Evol. 5(1), 135–141. doi: 10.1038/s41559-020-01331-w
107. Lhomond, B., and Saurel-Cubizolles, M. J. (2006). Violence against women and suicide risk: the neglected impact of same-sex sexual behaviour. Social Science and Medicine, 62(8), 2002-2013. doi: 10.1016/j.socscimed.2005.08.026
108. Lin, C., Moore, D. D., Nylund, D., and Espinoza, S. A. (2020). Clinical issues among Chinese gay men in counseling. J LGBT Issues Couns. 14(1), 18–37. doi: 10.1080/15538605.2020.1711290
109. Lin, K., and Wang, W. (2021). Changing public tolerance for same-sex sexual behaviors in China, 2010–2017: a decomposition analysis. Arch. Sex. 50(8), 3433–3445. [doi: 10.1007/s10508-021-02080-y](https://doi.org/10.1007/s10508-021-02080-y)
110. Liu, T., Dartevelle, L., Yuan, C., Wei, H., Wang, Y., Ferveur, J.-F., and Guo, A. (2008). Increased dopamine level enhances male-male courtship in Drosophila. J. Neurosci. 28, 5539–5546. doi: 10.1523/JNEUROSCI.5290-07.2008
111. Liu, T., Dartevelle, L., Yuan, C., Wei, H., Wang, Y., Ferveur, J.F., and Guo, A. (2009). Reduction of dopamine level enhances the attractiveness of male Drosophila to other males. PLoS One 4, 1–5. doi: 10.1371/journal.pone.0004574
112. Liu, T., Wang, Y., Tian, Y., Zhang, J., Zhao, J., and Guo, A. (2020). The receptor channel formed by ppk25, ppk29 and ppk23 can sense the *Drosophila* female pheromone 7,11-heptacosadiene. Genes Brain Behav. 19(2), 1–10. doi: 10.1111/gbb.12529
113. Liu, Z., Li, X., Prasifka, J.R., Jurenka, R., and Bonning, B.C. (2008). Overexpression of Drosophila juvenile hormone esterase binding protein results in anti-JH effects and reduced pheromone abundance. Gen. Comp. Endocrinol. 156, 164–172. doi: 10.1016/j.ygcen.2008.01.006
114. Logue, D.M., Mishra, S., McCaffrey, D., Ball, D., and Cade, W.H. (2009). A behavioral syndrome linking courtship behavior toward males and females predicts reproductive success from a single mating in the hissing cockroach, *Gromphadorhina portentosa*. Behav. Ecol. 20, 781–788. doi: 10.1093/beheco/arp061
115. London, A. S., VanLandingham, M. J., and Grandjean, N. (1997). Socio-demographic correlates, HIV/AIDS related cofactors, and measures of same-sex sexual behaviour among Northern Thai male soldiers. Health Transition Review, 33-60. doi:
116. Macchiano, A., Razik, I., and Sagot, M. (2018). Same-sex courtship behaviors in male-biased populations: evidence for the mistaken identity hypothesis. Acta Ethol. 21(3), 147–151. doi: 10.1007/s10211-018-0293-8
117. Macey, P., Morris, N., Hamlin, O., and Cravens, J. (2017). Bi-invisibility: a phenomenological exploration of same-sex casual encounters. J. Bisex. 17(2), 225–250. doi: 10.1080/15299716.2017.1296801
118. MacFarlane, G.R., Blomberg, S.P., and Vasey, P.L. (2010). Homosexual behaviour in birds: frequency of expression is related to parental care disparity between the sexes. Anim. Behav. 80, 375–390. doi: 10.1016/j.anbehav.2010.05.009
119. MacFarlane, G.R., Blomberg, S.P., Kaplan, G., and Rogers, L.J. (2007). Same-sex sexual behavior in birds: Expression is related to social mating system and state of development at hatching. Behav. Ecol. 18, 21–33. doi: 10.1093/beheco/arl065
120. Manji, A., Pena, R., and Dubrow, R. (2007). Sex, condoms, gender roles, and HIV transmission knowledge among adolescents in León, Nicaragua: Implications for HIV prevention. AIDS care. 19(8), 989-995. doi: 10.1080/09540120701244935
121. Marshal, M. P., Friedman, M. S., Stall, R., and Thompson, A. L. (2009). Individual trajectories of substance use in lesbian, gay and bisexual youth and heterosexual youth. Addiction. 104(6), 974-981. doi: 10.1111/j.1360-0443.2009.02531.x
122. Martin-Storey, A. (2015). Prevalence of dating violence among sexual minority youth: Variation across gender, sexual minority identity and gender of sexual partners. Journal of Youth and Adolescence. 44(1), 211-224. doi: 10.1007/s10964-013-0089-0
123. McCabe, S. E., Hughes, T. L., Bostwick, W. B., West, B. T., and Boyd, C. J. (2009). Sexual orientation, substance use behaviors and substance dependence in the United States. Addiction. 104(8), 1333-1345. doi: 10.1016/j.jsat.2012.01.007
124. McGregor, A. J., Bogart, L. M., Higgins-Biddle, M., Strolovitch, D. Z., and Ojikutu, B. (2019). Marginalized yet mobilized: race, sexuality, and the role of political hypervigilance in African American political participation in 2016. Du Bois Review. 16(1), 131–156. doi: 10.1017/S1742058X19000031
125. Meissner, G.W., Manoli, D.S., Chavez, J.F., Knapp, J.-M., Lin, T.L., Stevens, R.J., Mellert, D.J., Tran, D.H., and Baker, B.S. (2011). Functional dissection of the neural substrates for sexual behaviors in *Drosophila melanogaster*. Genetics 189, 195–211. doi: 10.1534/genetics.111.129940
126. Meng, X. J., Grulich, A., Wang, X. W., Yin, H. L., Gu, J., Zhang, X., Gu, J., and Zou, H. C. (2018). Repeat HIV testing and incident rates among individuals attending voluntary counseling and testing clinics in Wuxi, China: a retrospective study. Biomed. Envirom. Sci. 31(1), 37–47. doi: 10.3967/bes2018.004
127. Mizumoto, N., Yashiro, T., and Matsuura, K. (2016). Male same-sex pairing as an adaptive strategy for future reproduction in termites. Anim. Behav. 119, 179–187. doi: 10.1016/j.anbehav.2016.07.007
128. Monk, J. D., Giglio, E., Kamath, A., Lambert, M. R., and McDonough, C. E. (2020). Author Correction: An alternative hypothesis for the evolution of same-sex sexual behaviour in animals. Nat. Ecol. Evol. 4(1), 169. doi: 10.1038/s41559-019-1064-2
129. Monk, J. D., Giglio, E., Kamath, A., Lambert, M. R., and McDonough, C. E. (2019). An alternative hypothesis for the evolution of same-sex sexual behaviour in animals. Nat. Ecol. Evol. 3(12), 1622–1631. doi: 10.1038/s41559-019-1019-7
130. Mooijman, M., and Stern, C. (2016). When perspective taking creates a motivational threat: the case of conservatism, same-sex sexual behavior, and anti-gay attitudes. Pers. Soc. Psychol. 42(6), 738–754. doi: 10.1177/0146167216636633
131. Moscovice, L. R., Surbeck, M., Fruth, B., Hohmann, G., Jaeggi, A. V., and Deschner, T. (2019). The cooperative sex: Sexual interactions among female bonobos are linked to increases in oxytocin, proximity and coalitions. Horm and Behav. 116(March), 104581. doi: 10.1016/j.yhbeh.2019.104581
132. Murakami, S., and Itoh, M.T. (2003). Removal of both antennae influences the courtship and aggressive behaviors in male crickets. J. Neurobiol. 57, 110–118. doi: 10.1002/neu.10255
133. Murray, S. O. (2003). Representations of desires in some recent gay Asian-American writings. J Homosex. 45(1), 111-142. doi: 10.1300/J082v45n01_06
134. Muscarella, F. (2000). The evolution of homoerotic behavior in humans. J Homosex. 40(1), 51-77. doi: 10.1300/J082v40n01_03
135. Oliveira, R.F., and Almada, V.C. (1998). Mating tactics and male-male courtship in the lek-breeding cichlid *Oreochromis mossambicus*. J. Fish 52, 1115–1129. doi: 10.1111/j.1095-8649.1998.tb00959.x
136. Pampati, S., Lowry, R., and Steiner, R. J. (2019). Substance use, violence experiences, and mental health: Are these health risks associated with HIV testing among sexually experienced U.S. high school students? AIDS Care. 31(9), 1106–1113. doi: 10.1080/09540121.2019.1619666.Substance
137. Perales, F., Campbell, A. K., Everett, B. G., McNair, R., and Hughes, T. L. (2021). Prevalence and sociodemographic correlates of identifying as mainly heterosexual: stability and change across three cohorts of australian women. Arch. Sex. 50(8), 3459–3477. doi: 10.1007/s10508-021-02000-0
138. Pfau, D. R., Jordan, C. L., and Breedlove, S. M. (2021). Response to commentaries: Sniffing out consensus on the evolution of primate same-sex sexual behavior. Arch. Sex. 50(6), 2317–2320. doi: 10.1007/s10508-021-02078-6
139. Pfau, D., Jordan, C. L., and Breedlove, S. M. (2021). The de-scent of sexuality: did loss of a pheromone signaling protein permit the evolution of same-sex sexual behavior in primates? Arch. Sex. 50(6), 2267–2276. doi: 10.1007/s10508-018-1377-2
140. Pfaus, J. G. (2021). TRPC2: A pheromonal funnel into same-sex sexual behavior. Arch. Sex. 50(6), 2299–2300. doi: 10.1007/s10508-020-01699-7
141. Pham, J. M. (2019). Institutional, subcultural, and individual determinants of same-sex sexual contact among college women. Journal of Sex Research. 56(8), 1031–1044. doi: 10.1080/00224499.2019.1607239
142. Phillips, G., Beach, L. B., Turner, B., Feinstein, B. A., Marro, R., Philbin, M. M., Salamanca, P., Felt, D., and Birkett, M. (2019). Sexual identity and behavior among U.S. high school students, 2005–2015. Arch. Sex. 48(5), 1463–1479. doi: 10.1007/s10508-019-1404-y
143. Phillips, R. E., Kalp, D., Lucci, M., Maccarelli, A., Avant, S., Cenkner, D., and Herndon, R. (2017). Initial Validation of measures of sanctification in same-sex romantic relationships and sexual behavior. Journal for the Scientific Study of Religion. 56(4), 836–851. doi: 10.1111/jssr.12488
144. Pincemy, G., Dobson, F.S., and Jouventin, P. (2010). Homosexual mating displays in penguins. Ethology 116, 1210–1216. doi: 10.1111/j.1439-0310.2010.01835.x
145. Pingel, E. S., Thomas, L., Harmell, C., and Bauermeister, J. A. (2013). Creating comprehensive, youth centered, culturally appropriate sex education: What do young gay, bisexual, and questioning men want? Sexuality Research and Social Policy. 10(4), 293-301. doi: 10.1007/s13178-013-0134-5
146. Pivnick, K.A., Lavoir-Dornik, J., and McNeil, J. (1992). The role of the androconia in the mating behaviour of the European skipper, *Thymelicus lineola*, and evidence for a male sex pheromone. Physiol. Entomol. 17, 260–268. doi: 10.1111/j.1365-3032.1992.tb01020.x
147. Rasmussen, D. R. (1984). Functional alterations in the social organization of bonnet macaques (Macaca radiata) induced by ovariectomy: an experimental analysis. Psychoneuroendocrinology. 9(4), 343-374. doi: 10.1016/0306-4530(84)90043-X
148. Ray, T. N., and Parkhill, M. R. (2021). Heteronormativity, Disgust sensitivity, and hostile attitudes toward gay men: potential mechanisms to maintain social hierarchies. Sex Roles. 84(1–2), 49–60. [doi: 10.1007/s11199-020-01146-w](https://doi.org/10.1007/s11199-020-01146-w)
149. Rayner, J. G., and Bailey, N. W. (2019). Testing the role of same-sex sexual behaviour in the evolution of alternative male reproductive phenotypes. Anim. Behav. 157, 5–11. doi: 10.1016/j.anbehav.2019.08.017
150. Reed, L. I. (2010). Sexual orientation in males and the evolution of anisogamy. Medical hypotheses. 74(2), 261-263. doi: 10.1016/j.mehy.2009.09.019
151. Reinisch, J. M., Mortensen, E. L., and Sanders, S. A. (2017). Prenatal exposure to progesterone affects sexual orientation in humans. Arch. Sex. 46(5), 1239–1249. doi: 10.1007/s10508-016-0923-z
152. Rhodes, S. D., Hergenrather, K. C., Wilkin, A., Alegría-Ortega, J., and Montaño, J. (2006). Preventing HIV infection among young immigrant Latino men: results from focus groups using community-based participatory research. Journal of the National Medical Association, 98(4), 564. doi: 10.1006/pmed.2002.1047
153. Riccucci, M. (2011). Same-sex sexual behaviour in bats. Hystrix 22, 139–147. doi: 10.4404/Hystrix-22.1-4478
154. Rind, B. (2015). Response to Commentaries: Transgenerational Same-Sex Sexual Behavior, Typology, and Etiology. International Journal of Sexual Health. 27(3), 220-223. doi: 10.1080/19317611.2015.1066474
155. Rupp, L. J., Taylor, V., Regev-Messalem, S., Fogarty, A. C., and England, P. (2014). Queer women in the hookup scene: Beyond the closet? Gender and Society. 28(2), 212-235. doi: 10.1177/0891243213510782
156. Sales, K., Trent, T., Gardner, J., Lumley, A. J., Vasudeva, R., Michalczyk, Ł., Martin, O. Y., and Gage, M. J. G. (2018). Experimental evolution with an insect model reveals that male homosexual behaviour occurs due to inaccurate mate choice. Anim. Behav. *139*, 51–59. doi: 10.1016/j.anbehav.2018.03.004
157. Sandel, A. A., and Reddy, R. B. (2021). Sociosexual behaviour in wild chimpanzees occurs in variable contexts and is frequent between same-sex partners. Behaviour. 158(3–4), 249–276. doi: 10.1163/1568539X-bja10062
158. Sandfort, T. G., de Graaf, R., Bijl, R. V., and Schnabel, P. (2001). Same-sex sexual behavior and psychiatric disorders: Findings from the Netherlands Mental Health Survey and Incidence Study (NEMESIS). Archives of general psychiatry. 58(1), 85-91. doi: 10.1001/archpsyc.58.1.85
159. Sandnabba, N. K., and Ahlberg, C. (1999). Parents' attitudes and expectations about children's cross-gender behavior. Sex roles. 40(3), 249-263. doi: 10.1023/A:1018851005631
160. Savulescu, J., Earp, B. D., and Schuklenk, U. (2021). Ethics of genetic research on same-sex sexual behaviour. Nature Human Behaviour. 5(9), 1123–1124. [doi: 10.1038/s41562-021-01164-y](https://doi.org/10.1038/s41562-021-01164-y)
161. Scharf, I., and Martin, O.Y. (2013). Same-sex sexual behavior in insects and arachnids: prevalence, causes, and consequences. Behav. Ecol. Sociobiol. 67, 1719–1730. doi: 10.1007/s00265-013-1610-x
162. Schmitt, C. A., and Garrett, E. C. (2021). De-scent with modification: more evidence and caution needed to assess whether the loss of a pheromone signaling protein permitted the evolution of same-sex sexual behavior in primates. Arch. Sex. 50(6), 2301–2307. doi: 10.1007/s10508-019-01583-z
163. Sellami, A., Wegener, C., and Veenstra, J.A. (2012). Functional significance of the copper transporter ATP7 in peptidergic neurons and endocrine cells in *Drosophila melanogaster*. FEBS Lett. 586, 3633–3638. doi: 10.1016/j.febslet.2012.08.009
164. Shine, R., Harlow, P., LeMaster, M., Moore, I., and Mason, R. (2000). The transvestite serpent: why do male garter snakes court (some) other males? Anim. Behav. 59. 349–359. doi: 10.1006/anbe.1999.1321
165. Silva, V., Palacios-Muñoz, A., Volonté, M., Frenkel, L., Ewer, J., and Ons, S. (2021). Orcokinin neuropeptides regulate reproduction in the fruit fly, *Drosophila melanogaster*. Insect Biochemistry and Molecular Biology. *139*(September 2020). [doi: 10.1016/j.ibmb.2021.103676](https://doi.org/10.1016/j.ibmb.2021.103676)
166. Singh, D., Fine, D. N., and Marrazzo, J. M. (2011). Chlamydia trachomatis infection among women reporting sexual activity with women screened in Family Planning Clinics in the Pacific Northwest, 1997 to 2005. American journal of public health. 101(7), 1284-1290. doi: 10.2105/AJPH.2009.169631
167. Skinner, B. (2016). Mathematical toy model inspired by the problem of the adaptive origins of the sexual orientation continuum. R. Soc. Open Sci. 3(9). doi: 10.1098/rsos.160403
168. Songvorawit, N., Butcher, B. A., and Chaisuekul, C. (2019). Size does not matter: same-sex sexual behavior occurred regardless of mandible size in male stag beetle *Aegus chelifer chelifer* (Coleoptera: Lucanidae). Journal of Insect Behavior. 32(4–6), 282–289. doi: 10.1007/s10905-019-09733-w
169. Steinke, J., Root-Bowman, M., Estabrook, S., Levine, D. S., and Kantor, L. M. (2017). Meeting the needs of sexual and gender minority youth: formative research on potential digital health interventions. Journal of Adolescent Health. 60(5), 541–548. doi: 10.1016/j.jadohealth.2016.11.023
170. Stewart, J. L., Spivey, L. A., Widman, L., Choukas-Bradley, S., and Prinstein, M. J. (2019). Developmental patterns of sexual identity, romantic attraction, and sexual behavior among adolescents over three years. Journal of Adolescence. 77:90–97. doi: 10.1016/j.adolescence.2019.10.006
171. Sureau, G., and Ferveur, J. F. (1999). Co-adaptation of pheromone production and behavioural responses in Drosophila melanogaster males. Genetics Research. 74(2), 129-137. doi: 10.1017/S0016672399003936
172. Thompson, H. L., and Gordon, N. M. (2021). First description of nesting behavior of a same-sex pair of whooping cranes (*Grus americana*) in the reintroduced eastern migratory population. Waterbirds. *43*(3–4), 326–332. doi: 10.1675/063.043.0312
173. Toda, H., Zhao, X., and Dickson, B.J. (2012). The Drosophila female aphrodisiac pheromone activates ppk23+ sensory neurons to elicit male courtship behavior. Cell Rep. 1, 599–607. doi: 10.1016/j.celrep.2012.05.007
174. Todoroki, Y., Mochizuki, K., and Numata, H. (2015). Sexual attractiveness shared by both sexes mediates same‐sex sexual behaviour in the parasitoid wasp T elenomus triptus. Physiological Entomology. 40(3), 239-246. doi: 10.1111/phen.12107
175. Twenge, J. M., Sherman, R. A., and Wells, B. E. (2016). Changes in American Adults’ Reported Same-Sex Sexual Experiences and Attitudes, 1973–2014. Arch. Sex. *45*(7), 1713–1730. [doi: 10.1007/s10508-016-0769-4](https://doi.org/10.1007/s10508-016-0769-4)
176. Ungerfeld, R., and González-Pensado, S. P. (2008). Social rank affects reproductive development in male lambs. Animal reproduction science. 109(1-4), 161-171. doi: 10.1016/j.anireprosci.2007.12.006
177. Ungerfeld, R., Giriboni, J., Freitas-de-Melo, A., and Lacuesta, L. (2014). Homosexual behavior in male goats is more frequent during breeding season and in bucks isolated from females. Horm. Behav. 65, 516–520. doi: 10.1016/j.yhbeh.2014.04.013
178. Ungerfeld, R., Ramos, M.A., and Bielli, A. (2007). Relationship between male-male and male-female sexual behavior in 5-6-month-old male lambs. Anim. Reprod. Sci. 100, 385–390. doi: 10.1016/j.anireprosci.2006.09.022
179. Vasey, P. (2004). Same-sex sexual behavior and sexual partner preference in female Japanese macaques: behavioral and neuroanatomical research. Horm Beh. 46(1). doi:
180. Vasey, P. L., and Jiskoot, H. (2010). The biogeography and evolution of female homosexual behavior in Japanese macaques. Arch. Sex. 39(6), 1439-1441. doi: 10.1007/s10508-009-9518-2
181. Ventura-Aquino, E., and Paredes, R. G. (2021). Pheromones and same-sex sexual behavior. Arch. Sex. 50(6), 2309–2311. doi: 10.1007/s10508-020-01690-2
182. Vijayan, V., Thistle, R., Liu, T., Starostina, E., and Pikielny, C.W. (2014). Drosophila pheromone-sensing neurons expressing the ppk25 ion channel subunit stimulate male courtship and female receptivity. PLoS Genet. 10. doi: 10.1371/journal.pgen.1004238
183. Walker, J. N. J., and Longmire-Avital, B. (2013). The impact of religious faith and internalized homonegativity on resiliency for black lesbian, gay, and bisexual emerging adults. Dev. Psychol. 49(9), 1723. doi: 10.1037/a0031059
184. Wang, K., Guo, Y., Wang, F., and Wang, Z. (2011a). Drosophila TRPA channel painless inhibits male-male courtship behavior through modulating olfactory sensation. PLoS One 6. doi: 10.1371/journal.pone.0025890
185. Wang, L., Han, X., Mehren, J., Hiroi, M., Billeter, J.-C., Miyamoto, T., Amrein, H., Levine, J.D., and Anderson, D.J. (2011b). Hierarchical chemosensory regulation of male-male social interactions in Drosophila. Nat. Neurosci. 14, 757–762. doi: 10.1038/nn.2800
186. Wang, Q., Chen, L., Li, J., and Yin, X. (1996). Mating behavior of *Phytoecia rufiventris* Gautier (Coleoptera: Cerambycidae). J. Insect Behav. 9, 47–60. doi: 10.1007/BF02213723
187. Wells, J. E., McGee, M. A., and Beautrais, A. L. (2011). Multiple aspects of sexual orientation: Prevalence and sociodemographic correlates in a New Zealand national survey. Arch. Sex. 40(1), 155-168. doi: 10.1007/s10508-010-9636-x
188. Weng, R., Chin, J.S.R., Yew, J.Y., Bushati, N., and Cohen, S.M. (2013). miR-124 controls male reproductive success in Drosophila. Elife 2013, 1–16. doi: 10.7554/eLife.00640
189. Wichstrøm, L., and Hegna, K. (2003). Sexual orientation and suicide attempt: a longitudinal study of the general Norwegian adolescent population. Journal of abnormal psychology. 112(1), 144. doi: 10.1037/0021-843X.112.1.144
190. Xia, R. Y., Li, M. Q., Wu, Y. S., Qi, Y. X., Ye, G. Y., and Huang, J. (2016). A new family of insect muscarinic acetylcholine receptors. Insect Molecular Biology. *25*(4), 362–369. doi: 10.1111/imb.12229
191. Xie, Y., and Peng, M. (2018). Attitudes Toward Homosexuality in China: Exploring the Effects of Religion, Modernizing Factors, and Traditional Culture. J Homosex. *65*(13), 1758–1787. doi: 10.1080/00918369.2017.1386025
192. Xu, F., Sternberg, M. R., and Markowitz, L. E. (2010). Women who have sex with women in the United States: Prevalence, sexual behavior and prevalence of herpes simplex virus type 2 infection—Results from National Health and Nutrition Examination Survey 2001–2006. Sexually transmitted diseases. 37(7), 407-413. doi: 10.1097/OLQ.0b013e3181db2e18
193. Yamamoto, S., and Seto, E.S. (2014). Dopamine dynamics and signaling in Drosophila: an overview of genes, drugs and behavioral paradigms. Exp. Anim. 63, 107–119.
194. Yan, Y., Ziemek, J., and Schetelig, M. F. (2020). CRISPR/Cas9 mediated disruption of the white gene leads to pigmentation deficiency and copulation failure in *Drosophila suzukii*. Journal of Insect Physiology. 126(February), 104091. [doi: 10.1016/j.jinsphys.2020.104091](https://doi.org/10.1016/j.jinsphys.2020.104091)
195. Yang, X., Attané, I., Li, S., and Yang, B. (2012). Same-sex sexual behaviors among male migrants in a context of male “marriage squeeze” results from an exploratory survey in urban Xi’an, China. American journal of men's health. 6(6), 485-496. doi: 10.1177/1557988312453479
196. Yang, X., Attané, I., Li, S., and Zhang, Q. (2012). On same-sex sexual behaviors among male bachelors in rural China: Evidence from a female shortage context. American journal of men's health. 6(2), 108-119. doi: 10.1177/1557988311415512
197. Zhan, H., Li, D., Dewer, Y., Niu, C., Li, F., and Luo, C. (2021). Identification and functional characterization of odorant-binding proteins 69a and 76a of *Drosophila suzukii*. Heliyon. 7(3), e06427. doi: 10.1016/j.heliyon.2021.e06427
198. Zhang, S.D., Odenwald, W.F., Odenwald, F., Zhang, S.D., Odenwald, W.F., Odenwald, F., Zhang, S.D., and Odenwald, W.F. (1995). Misexpression of the white (w) gene triggers male-male courtship in Drosophila. Proc. Natl. Acad. Sci. U. S. A. 92, 5525–5529. doi: 10.1073/pnas.92.12.5525
199. Zietsch, B. P. (2021). More evidence and context are needed to evaluate the possibility that scent perception is part of the same-sex sexual behavior story. Arch. Sex. *50*(6), 2313–2315. doi: 10.1007/s10508-019-01562-4
200. Zietsch, B. P., Sidari, M. J., Abdellaoui, A., Maier, R., Långström, N., Guo, S., Beecham, G. W., Martin, E. R., Sanders, A. R., and Verweij, K. J. H. (2021). Genomic evidence consistent with antagonistic pleiotropy may help explain the evolutionary maintenance of same-sex sexual behaviour in humans. Nature Human Behaviour. *5*(9), 1251–1258. [doi: 10.1038/s41562-021-01168-8](https://doi.org/10.1038/s41562-021-01168-8)
201. Zietsch, B. P., Sidari, M. J., Abdellaoui, A., Maier, R., Långström, N., Guo, S., Beecham, G. W., Martin, E. R., Sanders, A. R., and Verweij, K. J. H. (2021). Erratum: Author Correction: Genomic evidence consistent with antagonistic pleiotropy may help explain the evolutionary maintenance of same-sex sexual behaviour in humans (Nature human behaviour (2021) 5 9 (1251-1258)). Nature Human Behaviour. *5*(9), 1259. doi: 10.1038/s41562-021-01210-9

### References S1.2. List of excluded references during screening.

1. Adriaens, P. R., and De Block, A. (2006). The evolution of a social construction: the case of male homosexuality. Perspectives in biology and medicine. 49(4), 570-585. doi: 10.1353/pbm.2006.0051
2. Alanko, K., Santtila, P., Witting, K., Varjonen, M., Jern, P., Johansson, A., *et al*. N. (2009). Psychiatric symptoms and same-sex sexual attraction and behavior in light of childhood gender atypical behavior and parental relationships. Journal of Sex Research. 46(5), 494-504. doi: 10.1080/00224490902846487
3. Benvenga, S. (2005). Central hormonal regulation and dimorphism of arousal. International journal of andrology, 28, 18-22. doi: 10.1111/j.1365-2605.2005.00583.x
4. Bishop, M. D., Fish, J. N., Hammack, P. L., and Russell, S. T. (2020). Sexual identity development milestones in three generations of sexual minority people: a national probability sample. Dev. Psychol. 56(11), 2177–2193. doi: 10.1037/dev0001105
5. Blumberg, S. J., Cynamon, M. L., Osborn, L., and Olson, L. (2003). The impact of touch‐tone data entry on reports of HIV and STD risk behaviors in telephone interviews.
6. Boyer, C. Reyn, and Galupo, M.P. (2015). ‘Prove it!’ Same-sex performativity among sexual minority women and men. Psychology & Sexuality. 6.4, 357-368. doi: 10.1080/19419899.2015.1021372
7. Brewer, M. (2018). Good Ol’ country boys playin’ on the farm: online articulations of rural masculinity by men who have sex with men. Sex Cult. 22(2), 355–379. doi: 10.1007/s12119-017-9470-6
8. Button, D. M., and Worthen, M. G. (2014). General strain theory for LGBQ and SSB youth: The importance of intersectionality in the future of feminist criminology. Feminist criminology. 9(4), 270-297. doi: 10.1177/1557085114525988
9. Caballero-Mendieta, N., and Cordero, C. (2012). Enigmatic liaisons in Lepidoptera: A review of same-sex courtship and copulation in butterflies and moths. Journal of Insect Science. 12(1), 138.
10. Cardoso, F. L. (2009). Similar faces of same-sex sexual behavior: a comparative ethnographical study in Brazil, Turkey, and Thailand. Journal of Homosexuality. 56(4), 457-484. doi: 10.1080/00918360902816866
11. Chae, D., Krieger, N., Bennett, G., Lindsey, J., and Stoddard, A. E. Barbeau. 2010. “Implications of Discrimination Based on Sexuality, Gender, and Race/Ethnicity for Psychological Distress Among Working-Class Sexual Minorities: The United for Health Study, 2003–2004.” Work and Health. 40(4), 589-608. doi: 10.2190/HS.40.4.b
12. Ciani, A. C., and Battaglia, U. (2014). Implicit measurements of sexual preference in self‐declared heterosexual men: A pilot study on the rate of androphilia in Italy. The journal of sexual medicine. 11(9), 2207-2217. doi: 10.1111/jsm.12565
13. Clancy, L. M., Cooper, A. L., Griffith, G. W., and Santer, R. D. (2017). Increased male-male mounting behaviour in desert locusts during infection with an entomopathogenic fungus. Sci. Rep. 7(1), 1–7. doi: 10.1038/s41598-017-05800-4
14. Clark, J. L., Caceres, C. F., Lescano, A. G., Konda, K. A., Leon, S. R., Jones, F. R., *et al.* (2007). Prevalence of same-sex sexual behavior and associated characteristics among low-income urban males in Peru. PloS one. 2(8), e778. doi: 10.1371/journal.pone.0000778
15. Closson, E. F., Sivasubramanian, M., Mayer, K. H., Srivastava, A., Safren, S. A., Anand, V. R., *et al.* (2014). The other side of the bridge: exploring the sexual relationships of men who have sex with men and their female partners in Mumbai, India. Culture, health & sexuality. 16(7), 780-791. doi: 10.1080/13691058.2014.911960
16. de Almeida Neto, C., McFarland, W., Murphy, E. L., Chen, S., Nogueira, F. A. H., Mendrone Jr, A., *et al.* (2007). Risk factors for human immunodeficiency virus infection among blood donors in Sao Paulo, Brazil, and their relevance to current donor deferral criteria. Transfusion, 47(4), 608-614. doi: 10.1111/j.1537-2995.2007.01161.x
17. Dube, E. M. (2000). The role of sexual behavior in the identification process of gay and bisexual males. Journal of Sex Research. 37(2), 123-132.
18. Dunkle, K. L., Jewkes, R. K., Murdock, D. W., Sikweyiya, Y., and Morrell, R. (2013). Prevalence of consensual male–male sex and sexual violence, and associations with HIV in South Africa: A population-based cross-sectional study. PLoS medicine. 10(6), e1001472. doi: 10.1371/journal.pmed.1001472
19. DuRant, R. H., Krowchuk, D. P., and Sinal, S. H. (1998). Victimization, use of violence, and drug use at school among male adolescents who engage in same-sex sexual behavior. The Journal of pediatrics. 133(1), 113-118.
20. Eliason, M. J. (1993). AIDS-related stigma and homophobia: implications for nursing education. Nurse Educator. 18(6), 27-30. doi: 10.1097/00006223-199311000-00016
21. Eskin, M., Kaynak-Demir, H., and Demir, S. (2005). Same-sex sexual orientation, childhood sexual abuse, and suicidal behavior in university students in Turkey. Archives of sexual behavior. 34(2), 185-195. doi: 10.1007/s10508-005-1796-8
22. Faulkner, A. H., and Cranston, K. (1998). Correlates of same-sex sexual behavior in a random sample of Massachusetts high school students.American journal of public health. 88(2), 262-266. doi: 10.2105/AJPH.88.2.262
23. Frisell, T., Lichtenstein, P., Rahman, Q., and Långström, N. (2010). Psychiatric morbidity associated with same-sex sexual behaviour: influence of minority stress and familial factors. Psychological medicine. 40(2), 315-324. doi: 10.1017/S0033291709005996
24. Galea, J. T., Kinsler, J. J., Galan, D. B., Calvo, G., Sánchez, H., Leon, S. R., et al. (2015). Factors associated with visible anogenital warts among HIV-uninfected Peruvian men who have sex with men and transwomen: a cross-sectional study. Sexually transmitted diseases. 42(4), 202-207. doi: 10.1097/OLQ.0000000000000253
25. Ganna, A., Verweij, K. J. H., Nivard, M. G., Maier, R., Busch, A. S., Abdellaoui, A., Guo, S., Fah, J., Lichtenstein, P., Auton, A., Harris, K. M., Gary, W., Martin, E. R., Sanders, A. R., Perry, J. R. B., Benjamin, M., and Zietsch, B. P. (2019). Large-scale GWAS reveals insights into the genetic architecture of same-sex sexual behavior. Science. 365(6456), 1–22. doi: 10.1126/science.aat7693
26. Ganna, A., Verweij, K. J. H., Nivard, M. G., Maier, R., Wedow, R., Busch, A. S., Abdellaoui, A., Guo, S., Sathirapongsasuti, J. F., Team, M. R., Lichtenstein, P., Lundström, S., Långström, N., Auton, A., Kathleen Mullan, H., Beecham, G. W., Martin, E. R., Sanders, A. R., Perry, J. R. B., et al. (2021). Comment on “Large-scale GWAS reveals insights into the genetic architecture of same-sex sexual behavior.” Science. 371(6536), eaba5693. [doi: 10.1126/science.aba5693](https://doi.org/10.1126/science.aba5693)
27. Giasson, L. O., and Haddad, C. F. (2006). Social interactions in *Hypsiboas albomarginatus* (Anura: Hylidae) and the significance of acoustic and visual signals. Journal of Herpetology, 40(2), 171-180. doi: 10.1670/205-05A.1
28. Glick, S. N., and Golden, M. R. (2010). Persistence of racial differences in attitudes toward homosexuality in the United States. Journal of acquired immune deficiency syndromes (1999). 55(4), 516. doi: 10.1097/QAI.0b013e3181f275e0
29. Goisauf, M., Akyüz, K., and Martin, G. M. (2020). Moving back to the future of big data-driven research: reflecting on the social in genomics. Humanit. 7(1), 1–9. doi: 10.1057/s41599-020-00544-5
30. Guevara-Fiore, P., Stapley, J., and Watt, P. J. (2010). Mating effort and female receptivity: how do male guppies decide when to invest in sex? Behavioral ecology and sociobiology. 64(10), 1665-1672. doi: 10.1007/s00265-010-0980-6
31. Hamer, D., Mustanski, B., Sell, R., Sanders, S. A., and Garcia, J. R. (2021). Comment on “Large-scale GWAS reveals insights into the genetic architecture of same-sex sexual behavior.” Science. 371(6536), eaba2941. doi: 10.1126/science.aba2941
32. Herbenick, D., Reece, M., Schick, V., Sanders, S. A., Dodge, B., and Fortenberry, J. D. (2010). An event‐level analysis of the sexual characteristics and composition among adults ages 18 to 59: Results from a national probability sample in the United States. The Journal of Sexual Medicine. 7, 346-361. doi: 10.1111/j.1743-6109.2010.02012.x
33. Herek, G. M. (1999). AIDS and stigma. American behavioral scientist. 42(7), 1106-1116. doi: 10.1177/0002764299042007006
34. Holloway, I. W., Padilla, M. B., Willner, L., and Guilamo-Ramos, V. (2015). Effects of minority stress processes on the mental health of Latino men who have sex with men and women: A qualitative study. Archives of sexual behavior. 44(7), 2087-2097. doi: 10.1007/s10508-014-0424-x
35. James, W. H. (2005). Biological and psychosocial determinants of male and female human sexual orientation. Journal of Biosocial Science. 37(5), 555. doi: 10.1017/S0021932004007059
36. Jeffery, A. J., Shackelford, T. K., Zeigler-Hill, V., Vonk, J., and Mcdonald, M. (2019). The evolution of human female sexual orientation introduction to human sexual orientation. 71–86. doi: 10.1007/s40806-018-0168-2
37. Kim, J. I., Lee, J. W., Lee, Y. A., Lee, D. H., Han, N. S., Choi, Y. K., *et al.* (2013). Sexual activity counteracts the suppressive effects of chronic stress on adult hippocampal neurogenesis and recognition memory. Brain research. 1538, 26-40. doi: 10.1016/j.brainres.2013.09.007
38. King, M., Green, J., Osborn, D. P., Arkell, J., Hetherton, J., and Pereira, E. (2005). Family size in white gay and heterosexual men. Archives of Sexual Behavior, 34(1), 117-122. doi: 10.1007/s10508-008-9386-1
39. Kuhle, B. X., and Radtke, S. (2013). Born both ways: The alloparenting hypothesis for sexual fluidity in women. Evolutionary Psychology. 11(2), 147470491301100202.
40. Kuperberg, A., and Walker, A. M. (2018). Heterosexual college students who hookup with same-sex partners. Arch. Sex. 47(5), 1387–1403. doi: 10.1007/s10508-018-1194-7
41. Lefevor, G. T., Sorrell, S. A., Kappers, G., Plunk, A., Schow, R. L., Rosik, C. H., and Beckstead, A. L. (2020). Same-Sex Attracted, Not LGBQ: The associations of sexual identity labeling on religiousness, sexuality, and health among mormons. J Homosex. 67(7), 940–964. doi: 10.1080/00918369.2018.1564006
42. Legate, N., and Rogge, R. D. (2019). Identifying Basic Classes of Sexual Orientation with Latent Profile Analysis: Developing the Multivariate Sexual Orientation Classification System. Arch. Sex, 48(5), 1403–1422. doi: 10.1007/s10508-018-1336-y
43. Lhomond, B., and Saurel-Cubizolles, M. J. (2006). Violence against women and suicide risk: the neglected impact of same-sex sexual behaviour. Social Science & Medicine. 62(8), 2002-2013. doi: 10.1016/j.socscimed.2005.08.026
44. Lin, C., Moore, D. D., Nylund, D., and Espinoza, S. A. (2020). Clinical issues among Chinese gay men in counseling. J LGBT Issues Couns. 14(1), 18–37. doi: 10.1080/15538605.2020.1711290
45. Lin, K., and Wang, W. (2021). Changing public tolerance for same-sex sexual behaviors in China, 2010–2017: a decomposition analysis. Arch. Sex. 50(8), 3433–3445. doi: 10.1007/s10508-021-02080-y
46. London, A. S., VanLandingham, M. J., and Grandjean, N. (1997). Socio-demographic correlates, HIV/AIDS related cofactors, and measures of same-sex sexual behaviour among Northern Thai male soldiers. Health Transition Review, 33-60.
47. Macey, P., Morris, N., Hamlin, O., and Cravens, J. (2017). Bi-invisibility: a phenomenological exploration of same-sex casual encounters. J. Bisex. 17(2), 225–250. doi: 10.1080/15299716.2017.1296801
48. Manji, A., Pena, R., and Dubrow, R. (2007). Sex, condoms, gender roles, and HIV transmission knowledge among adolescents in León, Nicaragua: Implications for HIV prevention. AIDS care, 19(8), 989-995. doi: 10.1080/09540120701244935
49. Marshal, M. P., Friedman, M. S., Stall, R., and Thompson, A. L. (2009). Individual trajectories of substance use in lesbian, gay and bisexual youth and heterosexual youth. Addiction. 104(6), 974-981. doi: 10.1111/j.1360-0443.2009.02531.x
50. Martin-Storey, A. (2015). Prevalence of dating violence among sexual minority youth: Variation across gender, sexual minority identity and gender of sexual partners. Journal of Youth and Adolescence. 44(1), 211-224. doi: 10.1007/s10964-013-0089-0
51. McCabe, S. E., Hughes, T. L., Bostwick, W. B., West, B. T., and Boyd, C. J. (2009). Sexual orientation, substance use behaviors and substance dependence in the United States. Addiction, 104(8), 1333-1345. doi: 10.1016/j.jsat.2012.01.007
52. McGregor, A. J., Bogart, L. M., Higgins-Biddle, M., Strolovitch, D. Z., and Ojikutu, B. (2019). Marginalized yet mobilized: race, sexuality, and the role of political hypervigilance in African American political participation in 2016. Du Bois Review. 16(1), 131–156. doi: 10.1017/S1742058X19000031
53. Meng, X. J., Grulich, A., Wang, X. W., Yin, H. L., Gu, J., Zhang, X., Gu, J., and Zou, H. C. (2018). Repeat HIV testing and incident rates among individuals attending voluntary counseling and testing clinics in Wuxi, China: a retrospective study. Biomed. Envirom. Sci. 31(1), 37–47. doi: 10.3967/bes2018.004
54. Mizumoto, N., Yashiro, T., and Matsuura, K. (2016). Male same-sex pairing as an adaptive strategy for future reproduction in termites. Anim. Behav. 119, 179–187. doi: 10.1016/j.anbehav.2016.07.007
55. Monk, J. D., Giglio, E., Kamath, A., Lambert, M. R., and McDonough, C. E. (2020). Author Correction: An alternative hypothesis for the evolution of same-sex sexual behaviour in animals. Nat. Ecol. Evol. 4(1), 169. doi: 10.1038/s41559-019-1064-2
56. Mooijman, M., and Stern, C. (2016). When perspective taking creates a motivational threat: the case of conservatism, same-sex sexual behavior, and anti-gay attitudes. Pers. Soc. Psychol. 42(6), 738–754. doi: 10.1177/0146167216636633
57. Moscovice, L. R., Surbeck, M., Fruth, B., Hohmann, G., Jaeggi, A. V., and Deschner, T. (2019). The cooperative sex: Sexual interactions among female bonobos are linked to increases in oxytocin, proximity and coalitions. Horm Behav. 116, 104581. doi: 10.1016/j.yhbeh.2019.104581
58. Murray, S. O. (2003). Representations of desires in some recent gay Asian-American writings. Journal of homosexuality. 45(1), 111-142. doi: 10.1300/J082v45n01_06
59. Muscarella, F. (2000). The evolution of homoerotic behavior in humans. Journal of Homosexuality. 40(1), 51-77. doi: 10.1300/J082v40n01_03
60. Pampati, S., Lowry, R., and Steiner, R. J. (2019). Substance use, violence experiences, and mental health: Are these health risks associated with HIV testing among sexually experienced U.S. high school students? AIDS Care. 31(9), 1106–1113. doi: 10.1080/09540121.2019.1619666.Substance
61. Perales, F., Campbell, A. K., Everett, B. G., McNair, R., and Hughes, T. L. (2021). Prevalence and sociodemographic correlates of identifying as mainly heterosexual: stability and change across three cohorts of australian women. Arch. Sex. 50(8), 3459–3477. doi: 10.1007/s10508-021-02000-0
62. Pfau, D. R., Jordan, C. L., and Breedlove, S. M. (2021). Response to commentaries: Sniffing out consensus on the evolution of primate same-sex sexual behavior. Arch. Sex. 50(6), 2317–2320. doi: 10.1007/s10508-021-02078-6
63. Pham, J. M. (2019). Institutional, subcultural, and individual determinants of same-sex sexual contact among college women. Journal of Sex Research. 56(8), 1031–1044. doi: 10.1080/00224499.2019.1607239
64. Phillips, G., Beach, L. B., Turner, B., Feinstein, B. A., Marro, R., Philbin, M. M., Salamanca, P., Felt, D., and Birkett, M. (2019). Sexual identity and behavior among U.S. high school students, 2005–2015. Arch. Sex. 48(5), 1463–1479. doi: 10.1007/s10508-019-1404-y
65. Phillips, R. E., Kalp, D., Lucci, M., Maccarelli, A., Avant, S., Cenkner, D., and Herndon, R. (2017). Initial Validation of measures of sanctification in same-sex romantic relationships and sexual behavior. Journal for the Scientific Study of Religion. 56(4), 836–851. doi: 10.1111/jssr.12488
66. Pingel, E. S., Thomas, L., Harmell, C., and Bauermeister, J. A. (2013). Creating comprehensive, youth centered, culturally appropriate sex education: What do young gay, bisexual, and questioning men want? Sexuality Research and Social Policy. 10(4), 293-301. doi: 10.1007/s13178-013-0134-5
67. Rasmussen, D. R. (1984). Functional alterations in the social organization of bonnet macaques (*Macaca radiata*) induced by ovariectomy: an experimental analysis. Psychoneuroendocrinology. 9(4), 343-374. doi: 10.1016/0306-4530(84)90043-X
68. Ray, T. N., and Parkhill, M. R. (2021). Heteronormativity, Disgust sensitivity, and hostile attitudes toward gay men: potential mechanisms to maintain social hierarchies. Sex Roles. 84(1–2), 49–60. doi: 10.1007/s11199-020-01146-w
69. Reinisch, J. M., Mortensen, E. L., and Sanders, S. A. (2017). Prenatal exposure to progesterone affects sexual orientation in humans. Arch. Sex. 46(5), 1239–1249. doi: 10.1007/s10508-016-0923-z
70. Rhodes, S. D., Hergenrather, K. C., Wilkin, A., Alegría-Ortega, J., and Montaño, J. (2006). Preventing HIV infection among young immigrant Latino men: results from focus groups using community-based participatory research. Journal of the National Medical Association. 98(4), 564. doi: 10.1006/pmed.2002.1047
71. Rind, B. (2015). Response to Commentaries: Transgenerational Same-Sex Sexual Behavior, Typology, and Etiology. International Journal of Sexual Health. 27(3), 220-223. doi: 10.1080/19317611.2015.1066474
72. Rupp, L. J., Taylor, V., Regev-Messalem, S., Fogarty, A. C., and England, P. (2014). Queer women in the hookup scene: Beyond the closet? Gender & Society. 28(2), 212-235. doi: 10.1177/0891243213510782
73. Sandfort, T. G., de Graaf, R., Bijl, R. V., and Schnabel, P. (2001). Same-sex sexual behavior and psychiatric disorders: Findings from the Netherlands Mental Health Survey and Incidence Study (NEMESIS). Archives of general psychiatry. 58(1), 85-91. doi: 10.1001/archpsyc.58.1.85
74. Sandnabba, N. K., and Ahlberg, C. (1999). Parents' attitudes and expectations about children's cross-gender behavior. Sex roles. 40(3), 249-263. doi: 10.1023/A:1018851005631
75. Singh, D., Fine, D. N., and Marrazzo, J. M. (2011). Chlamydia trachomatis infection among women reporting sexual activity with women screened in Family Planning Clinics in the Pacific Northwest, 1997 to 2005. American journal of public health. 101(7), 1284-1290. doi: 10.2105/AJPH.2009.169631
76. Steinke, J., Root-Bowman, M., Estabrook, S., Levine, D. S., and Kantor, L. M. (2017). Meeting the needs of sexual and gender minority youth: formative research on potential digital health interventions. Journal of Adolescent Health. 60(5), 541–548. doi: 10.1016/j.jadohealth.2016.11.023
77. Stewart, J. L., Spivey, L. A., Widman, L., Choukas-Bradley, S., and Prinstein, M. J. (2019). Developmental patterns of sexual identity, romantic attraction, and sexual behavior among adolescents over three years. Journal of Adolescence. 77:90–97. doi: 10.1016/j.adolescence.2019.10.006
78. Thompson, H. L., and Gordon, N. M. (2021). First description of nesting behavior of a same-sex pair of whooping cranes (*Grus americana*) in the reintroduced eastern migratory population. Waterbirds. *43*(3–4), 326–332. doi: 10.1675/063.043.0312
79. Twenge, J. M., Sherman, R. A., and Wells, B. E. (2016). Changes in American Adults’ Reported Same-Sex Sexual Experiences and Attitudes, 1973–2014. Arch. Sex. 45(7), 1713–1730. doi: 10.1007/s10508-016-0769-4
80. Vasey, P. (2004). Same-sex sexual behavior and sexual partner preference in female Japanese macaques: behavioral and neuroanatomical research. Hormones and Behavior. 46(1).
81. Vasey, P. L., and Jiskoot, H. (2010). The biogeography and evolution of female homosexual behavior in Japanese macaques. Archives of Sexual Behavior. 39(6), 1439-1441. doi: 10.1007/s10508-009-9518-2
82. Walker, J. N. J., and Longmire-Avital, B. (2013). The impact of religious faith and internalized homonegativity on resiliency for black lesbian, gay, and bisexual emerging adults. Developmental psychology. 49(9), 1723. doi: 10.1037/a0031059
83. Wells, J. E., McGee, M. A., and Beautrais, A. L. (2011). Multiple aspects of sexual orientation: Prevalence and sociodemographic correlates in a New Zealand national survey. Archives of Sexual Behavior. 40(1), 155-168. doi: 10.1007/s10508-010-9636-x
84. Wichstrøm, L., and Hegna, K. (2003). Sexual orientation and suicide attempt: a longitudinal study of the general Norwegian adolescent population. Journal of abnormal psychology. 112(1), 144. doi: 10.1037/0021-843X.112.1.144
85. Xie, Y., and Peng, M. (2018). Attitudes Toward Homosexuality in China: Exploring the Effects of Religion, Modernizing Factors, and Traditional Culture. J Homosex. 65(13), 1758–1787. doi: 10.1080/00918369.2017.1386025
86. Xu, F., Sternberg, M. R., and Markowitz, L. E. (2010). Women who have sex with women in the United States: Prevalence, sexual behavior and prevalence of herpes simplex virus type 2 infection—Results from National Health and Nutrition Examination Survey 2001–2006. Sexually transmitted diseases. 37(7), 407-413. doi: 10.1097/OLQ.0b013e3181db2e18
87. Yan, Y., Ziemek, J., and Schetelig, M. F. (2020). CRISPR/Cas9 mediated disruption of the white gene leads to pigmentation deficiency and copulation failure in Drosophila suzukii. Journal of Insect Physiology. 126(February), 104091. doi: 10.1016/j.jinsphys.2020.104091
88. Yang, X., Attané, I., Li, S., and Yang, B. (2012). Same-sex sexual behaviors among male migrants in a context of male “marriage squeeze” results from an exploratory survey in urban Xi’an, China. American journal of men's health. 6(6), 485-496. doi: 10.1177/1557988312453479
89. Yang, X., Attané, I., Li, S., and Zhang, Q. (2012). On same-sex sexual behaviors among male bachelors in rural China: Evidence from a female shortage context. American journal of men's health. 6(2), 108-119. doi: 10.1177/1557988311415512
90. Zietsch, B. P. (2021). More evidence and context are needed to evaluate the possibility that scent perception is part of the same-sex sexual behavior story. Arch. Sex. 50(6), 2313–2315. doi: 10.1007/s10508-019-01562-4
91. Zietsch, B. P., Sidari, M. J., Abdellaoui, A., Maier, R., Långström, N., Guo, S., Beecham, G. W., Martin, E. R., Sanders, A. R., and Verweij, K. J. H. (2021). Genomic evidence consistent with antagonistic pleiotropy may help explain the evolutionary maintenance of same-sex sexual behaviour in humans. Nature Human Behaviour. 5(9), 1251–1258. doi: 10.1038/s41562-021-01168-8
92. Zietsch, B. P., Sidari, M. J., Abdellaoui, A., Maier, R., Långström, N., Guo, S., Beecham, G. W., Martin, E. R., Sanders, A. R., and Verweij, K. J. H. (2021). Erratum: Author Correction: Genomic evidence consistent with antagonistic pleiotropy may help explain the evolutionary maintenance of same-sex sexual behaviour in humans (Nature human behaviour (2021) 5 9 (1251-1258)). Nature Human Behaviour. 5(9), 1259. doi: 10.1038/s41562-021-01210-9

### References S1.3. List of excluded references during eligibility.

1. Adkins-Regan, E. (2014). Male-male sexual behavior in Japanese quail: Being “on top” reduces mating and fertilization with females. Behavioural processes. 108, 71-79. doi: 10.1016/j.beproc.2014.09.027
2. Barron, A. B., and Hare, B. (2020). Prosociality and a sociosexual hypothesis for the evolution of same-sex attraction
3. Berger, D., You, T., Minano, M. R., Grieshop, K., Lind, M. I., Arnqvist, G., and Maklakov, A. A. (2016). Sexually antagonistic selection on genetic variation underlying both male and female same-sex sexual behavior. BMC Evol Biol. 16(88), 1–11. [doi: 10.1186/s12862-016-0658-4](https://doi.org/10.1186/s12862-016-0658-4)
4. Bierbach, D., Jung, C. T., Hornung, S., Streit, B., and Plath, M. (2013). Homosexual behaviour increases male attractiveness to females. Biology Letters. 9(1), 20121038. doi: 10.1098/rsbl.2012.1038
5. Blohowiak, C. C., Zelenka, D. J., and Siegel, P. B. (1985). Ontogeny of aggressive-sexual behavior between males in lines of Japanese quail (*Coturnix coturnix japonica*) selected for male mating frequency. Journal of Comparative Psychology. 99(1), 30. doi: 10.1037/0735-7036.99.1.30
6. Brooker, J. S., Webb, C. E., and Clay, Z. (2020). Fellatio among male sanctuary-living chimpanzees during a period of social tension. Behaviour. 158(1), 77–87. doi: 10.1163/1568539X-bja10053
7. Burgevin, L., Friberg, U., and Maklakov, A. A. (2013). Intersexual correlation for same-sex sexual behaviour in an insect. Animal Behaviour. 85(4), 759-762. doi: 10.1016/j.anbehav.2013.01.017
8. Chevalier-Skolnikoff, S. (1974). Male-female, female-female, and male-male sexual behavior in the stumptail monkey, with special attention to the female orgasm. Archives of sexual behavior. 3(2), 95-116. doi: 10.1007/BF01540994
9. Ciani, A. C., and Battaglia, U. (2014). Implicit measurements of sexual preference in self‐declared heterosexual men: A pilot study on the rate of androphilia in Italy. The journal of sexual medicine. 11(9), 2207-2217. doi: 10.1111/jsm.12565
10. Clive, J., Flintham, E., and Savolainen, V. (2020). Understanding same-sex sexual behaviour requires thorough testing rather than reinvention of theory. Nat. Ecol. Evol. 4(6), 784–785. doi: 10.1038/s41559-020-1189-3
11. Dickins, T. E., and Rahman, Q. (2020). Ancestral primacy of same-sex sexual behaviour does not explain its stable prevalence in modern populations. Nat. Ecol. Evol. 4(6), 782–783. [doi: 10.1038/s41559-020-1187-5](https://doi.org/10.1038/s41559-020-1187-5)
12. Engel, K. C., Männer, L., Ayasse, M., and Steiger, S. (2015). Acceptance threshold theory can explain occurrence of homosexual behaviour. Biology letters. 11(1), 20140603. doi: 10.1098/rsbl.2014.0603
13. Genovart, M., Pradel, R., and Oro, D. (2012). Exploiting uncertain ecological fieldwork data with multi‐event capture–recapture modelling: an example with bird sex assignment. Journal of animal ecology. 81(5), 970-977. doi: 10.1111/j.1365-2656.2012.01991.x
14. Han, C. S., and Brooks, R. C. (2015). Same-sex sexual behaviour as a by-product of reproductive strategy under male–male scramble competition. Animal behaviour. 108, 193-197. doi: 10.1016/j.anbehav.2015.07.035
15. Han, C. S., and Brooks, R. C. (2015). The interaction between genotype and juvenile and adult density environment in shaping multidimensional reaction norms of behaviour. Functional Ecology. 29(1), 78-87. doi: 10.1111/1365-2435.12299
16. Han, C. S., Santostefano, F., and Dingemanse, N. J. (2016). Do social partners affect same-sex sexual behaviour in male water striders? Anim. Behav. 116, 53–59. doi: 10.1016/j.anbehav.2016.03.033
17. Hoving, H. J. T., Fernández-Álvarez, F., Portner, E. J., and Gilly, W. F. (2019). Same-sex sexual behaviour in an oceanic ommastrephid squid, *Dosidicus gigas* (Humboldt squid). Mar. Biol. 166(3), 1–7. [doi: 10.1007/s00227-019-3476-6](https://doi.org/10.1007/s00227-019-3476-6)
18. Hoving, H. J., Bush, S. L., and Robison, B. H. A shot in the dark: same-sex sexual behaviour in a deep-sea. doi: 10.1098/rsbl.2011.0680
19. Kamath, A., McDonough, C. E., Monk, J. D., Lambert, M. R., and Giglio, E. (2020). A. Kamath et al. reply. Nat. Ecol. Evol. 4(6), 786–787. doi: 10.1038/s41559-020-1188-4
20. Keller, M., and Chamero, P. (2021). Is the loss of Trpc2 gene function a real working hypothesis for the emergence of same-sex sexual behavior in Old World Primates? Arch. Sex. 50(6), 2293–2297. doi: 10.1007/s10508-020-01664-4
21. Leca, J. B., Gunst, N., and Vasey, P. L. (2014). Male homosexual behavior in a free-ranging all-male group of Japanese macaques at Minoo, Japan. Archives of Sexual Behavior. 43(5), 853-861. doi: 10.1007/s10508-014-0310-6
22. Lerch, B. A., and Servedio, M. R. (2021). Same-sex sexual behaviour and selection for indiscriminate mating. Nat. Ecol. Evol. 5(1), 135–141. doi: 10.1038/s41559-020-01331-w
23. Pfau, D., Jordan, C. L., and Breedlove, S. M. (2021). The de-scent of sexuality: did loss of a pheromone signaling protein permit the evolution of same-sex sexual behavior in primates? Arch. Sex. 50(6), 2267–2276. doi: 10.1007/s10508-018-1377-2
24. Pfaus, J. G. (2021). TRPC2: A pheromonal funnel into same-sex sexual behavior. Arch. Sex. 50(6), 2299–2300. doi: 10.1007/s10508-020-01699-7
25. Reed, L. I. (2010). Sexual orientation in males and the evolution of anisogamy. Medical hypotheses. 74(2), 261-263. doi: 10.1016/j.mehy.2009.09.019
26. Sales, K., Trent, T., Gardner, J., Lumley, A. J., Vasudeva, R., Michalczyk, Ł., Martin, O. Y., and Gage, M. J. G. (2018). Experimental evolution with an insect model reveals that male homosexual behaviour occurs due to inaccurate mate choice. Anim. Behav. 139, 51–59. doi: 10.1016/j.anbehav.2018.03.004
27. Sandel, A. A., and Reddy, R. B. (2021). Sociosexual behaviour in wild chimpanzees occurs in variable contexts and is frequent between same-sex partners. Behaviour. 158(3–4), 249–276. doi: 10.1163/1568539X-bja10062
28. Savulescu, J., Earp, B. D., and Schuklenk, U. (2021). Ethics of genetic research on same-sex sexual behaviour. Nature Human Behaviour. 5(9), 1123–1124. doi: 10.1038/s41562-021-01164-y
29. Schmitt, C. A., and Garrett, E. C. (2021). De-scent with modification: more evidence and caution needed to assess whether the loss of a pheromone signaling protein permitted the evolution of same-sex sexual behavior in primates. Arch. Sex. 50(6), 2301–2307. doi: 10.1007/s10508-019-01583-z
30. Sureau, G., and Ferveur, J. F. (1999). Co-adaptation of pheromone production and behavioural responses in Drosophila melanogaster males. Genetics Research. 74(2), 129-137. doi: 10.1017/S0016672399003936
31. Todoroki, Y., Mochizuki, K., and Numata, H. (2015). Sexual attractiveness shared by both sexes mediates same‐sex sexual behaviour in the parasitoid wasp *T elenomus triptus*. Physiological Entomology. 40(3), 239-246. doi: 10.1111/phen.12107
32. Ungerfeld, R., and González-Pensado, S. P. (2008). Social rank affects reproductive development in male lambs. Animal reproduction science. 109(1-4), 161-171. doi: 10.1016/j.anireprosci.2007.12.006
33. Ventura-Aquino, E., and Paredes, R. G. (2021). Pheromones and same-sex sexual behavior. Arch. Sex. 50(6), 2309–2311. doi: 10.1007/s10508-020-01690-2

### References S1.4. List of included references during screening.

1. Abbassi, P., and Burley, N.T. (2012). Nice guys finish last: Same-sex sexual behavior and pairing success in male budgerigars. Behav. Ecol. *23*, 775–782.
2. Adachi, H., and Soma, M. (2019). Vocalization can mediate male-male sexual interactions in Java sparrows. Anim. Biol. J, 69(1), 5–15. doi: 10.1163/15707563-00001051.
3. Agrawal, S., and Riffell, J.A. (2011). Behavioral neurobiology: the bitter life of male flies. Curr. Biol. *21*, R470–R472.
4. Amorim, M.C.P., Fonseca, P.J., and Almada, V.C. (2003). Sound production during courtship and spawning of *Oreochromis mossambicus*: male-female and male-male interactions. J. Fish Biol. *62*, 658–672.
5. Anaka, M., Anaka, M., Macdonald, C.D., Barkova, E., Simon, K., Rostom, R., Godoy, R. a., Haigh, A.J., Meinertzhagen, I. a., and Lloyd, V. (2008). The *white* Gene of *Drosophila* *melanogaster* encodes a protein with a role in courtship behavior. J. Neurogenet. *22*, 243–276.
6. Bailey, N.W., and French, N. (2012). Same-sex sexual behaviour and mistaken identity in male field crickets, *Teleogryllus oceanicus*. Anim. Behav. *84*.
7. Bailey, N.W., and Zuk, M. (2009). Same-sex sexual behavior and evolution. Trends Ecol. Evol. *24*, 439–446.
8. Bailey, N.W., Hoskins, J.L., Green, J., and Ritchie, M.G. (2013). Measuring same-sex sexual behaviour: the influence of the male social environment. Anim. Behav. *86*, 91–100.
9. Benelli, G., and Canale, A. (2013). Male-male sexual behavior in the parasitic wasp *Psyttalia concolor*. J. Insect Sci. *13*, 25.
10. Benelli, G., Bonsignori, G., Stefanini, C., and Canale, A. (2012). Courtship and mating behaviour in the fruit fly parasitoid *Psyttalia concolor* (Szépligeti) (Hymenoptera: Braconidae): the role of wing fanning. J. Pest Sci. (2004). *85*, 55–63.
11. Benelli, G., Bonsignori, G., Stefanini, C., Raspi, A., and Canale, A. (2013). The production of female sex pheromone in *Bactrocera oleae* (Rossi) young males does not influence their mating chances. Entomol. Sci. *16*, 47–53.
12. Bonnet, X., Golubović, A., Arsovski, D., Dević, S., Ballouard, J. M., Sterijovski, B., Ajtić, R., Barbraud, C., and Tomović, L. (2016). A prison effect in a wild population: a scarcity of females induces homosexual behaviors in males. Behav. Ecol. 27(4), 1206–1215. doi: 10.1093/beheco/arw023.
13. Boutin, S.R.T., Harrison, S.J., Fitzsimmons, L.P., McAuley, E.M., and Bertram, S.M. (2016). Same-sex sexual behaviour in crickets: understanding the paradox. Anim. Behav. *114*, 101–110.
14. Caballero-Mendieta, N., and Cordero, C. (2012). Enigmatic liaisons in Lepidoptera: a review of same-sex courtship and copulation in butterflies and moths. J. Insect Sci. *12*, 138.
15. Certel, S.J., Leung, A., Lin, C.Y., Perez, P., Chiang, A.S., and Kravitz, E.A. (2010). Octopamine neuromodulatory effects on a social behavior decision-making network in Drosophila males. PLoS One *5*.
16. Chan, Y.-B., and Kravitz, E.A. (2007). Specific subgroups of Fru(M) neurons control sexually dimorphic patterns of aggression in *Drosophila melanogaster*. Proc. Natl. Acad. Sci. U. S. A. *104*, 19577–19582.
17. Chen, B., Liu, H., Ren, J., and Guo, A. (2012). Mutation of Drosophila dopamine receptor DopR leads to male-male courtship behavior. Biochem. Biophys. Res. Commun. *423*, 557–563.
18. Chen, S. L., Chen, Y. H., Wang, C. C., Yu, Y. W., Tsai, Y. C., Hsu, H. W., Wu, C. L., Wang, P. Y., Chen, L. C., Lan, T. H., and Fu, T. F. (2017). Active and passive sexual roles that arise in Drosophila male-male courtship are modulated by dopamine levels in PPL2ab neurons. Sci. Rep. 7, 1–10. doi: 10.1038/srep44595.
19. Chertemps, T., François, A., Durand, N., Rosell, G., Dekker, T., Lucas, P., and Maïbèche-Coisne, M. (2012). A carboxylesterase, Esterase-6, modulates sensory physiological and behavioral response dynamics to pheromone in Drosophila. BMC Biol. *10*, 56.
20. Crossley, S.A., Bennet-Clark, H.C., and Evert, H.T. (1995). Courtship song components affect male and female Drosophila differently. Anim. Behav. *50*, 827–839.
21. Dai, H., Chen, Y., Chen, S., Mao, Q., Kennedy, D., Landback, P., Eyre-Walker, A., Du, W., and Long, M. (2008). The evolution of courtship behaviours through the origination of a new gene in Drosophila. Proc. Natl. Acad. Sci. U. S. A. *105*, 7478–7483.
22. Dalton, J.E., Lebo, M.S., Sanders, L.E., Sun, F., and Arbeitman, M.N. (2009). Ecdysone receptor acts in fruitless- expressing neurons to mediate Drosophila courtship behaviors. Curr. Biol. *19*, 1447–1452.
23. Deshpande, S. A., Meiselman, M., Hice, R. H., Arensburger, P., Rivera-Perez, C., Kim, D. H., Croft, R. L., Noriega, F. G., and Adams, M. E. (2019). Ecdysis triggering hormone receptors regulate male courtship behavior via antennal lobe interneurons in Drosophila. Gen. Comp. Endocrinol. 278(May 2018), 79–88. doi: 10.1016/j.ygcen.2018.12.003.
24. Dukas, R. (2010). Causes and consequences of male-male courtship in fruit flies. Anim. Behav. *80*, 913–919.
25. Eddison, M. (2021). A genetic screen for Drosophila social isolation mutants and analysis of sex pistol. Sci. Rep. 11(1). doi: 10.1038/s41598-021-96871-x.
26. Ejima, A. (2015). Pleiotropic actions of the male pheromone cis-vaccenyl acetate in *Drosophila melanogaster*. J. Comp. Physiol. A Neuroethol. Sensory, Neural, Behav. Physiol. *201*, 927–932.
27. Elie, J.E., Mathevon, N., and Vignal, C. (2011). Same-sex pair-bonds are equivalent to male-female bonds in a life-long socially monogamous songbird. Behav. Ecol. Sociobiol. *65*, 2197–2208.
28. Flintham, E. O., Yoshida, T., Smith, S., Pavlou, H. J., Goodwin, S. F., Carazo, P., and Wigby, S. (2018). Interactions between the sexual identity of the nervous system and the social environment mediate lifespan in Drosophila melanogaster. Proc. R. Soc. B: Biol. Sci. 285. doi: 10.1098/rspb.2018.1450.
29. Gaines, P., Tompkins, L., Woodard, C.T., and Carlson, J.R. (2000). quick-to-court, a Drosophila mutant with elevated levels of sexual behavior, is defective in a predicted coiled-coil protein. Genetics *154*, 1627–1637.
30. Ganter, G.K., Panaitiu, A.E., Desilets, J.B., Davis-Heim, J.A., Fisher, E.A., Tan, L.C.H., Heinrich, R., Buchanan, E.B., Brooks, K.M., Kenney, M.T., *et al.* (2011). Drosophila male courtship behavior is modulated by ecdysteroids. J. Insect Physiol. *57*, 1179–1184.
31. Ganter, G.K., Walton, K.L., Merriman, J.O., Salmon, M. V., Brooks, K.M., Maddula, S., and Kravitz, E.A. (2007). Increased male-male courtship in ecdysone receptor deficient adult flies. Behav. Genet. *37*, 507–512.
32. Giunti, G., Campolo, O., Laudani, F., and Palmeri, V. (2018). Male courtship behaviour and potential for female mate choice in the black soldier fly *Hermetia illucens* L. (Diptera: Stratiomyidae). Entomol. Gen. 38(1), 29–46. doi: 10.1127/entomologia/2018/0657.
33. Gupta, T., Morgan, H. R., Andrews, J. C., Brewer, E. R., and Certel, S. J. (2017). Methyl-CpG binding domain proteins inhibit interspecies courtship and promote aggression in Drosophila. Sci. Rep. 7(1), 1–12. doi: 10.1038/s41598-017-05844-6.
34. Hing, A.L., and Carlson, J.R. (1996). Male-male courtship behavior induced by ectopic expression of the Drosophila white gene: role of sensory function and age. J Neurobiol *30*, 454–464.
35. Hoskins, J.L., Ritchie, M.G., and Bailey, N.W. (2015). A test of genetic models for the evolutionary maintenance of same-sex sexual behaviour. Proc. R. Soc. B Biol. Sci. *282*, 20150429.
36. Isa, M., Kumano, N., and Tatsuta, H. (2019). When a male perceives a female: The effect of waxy components on the body surface on decision-making in the invasive pest weevil. R. Soc. Open Sci. 6(2). doi: 10.1098/rsos.181542.
37. Jankowiak, Ł., Tryjanowski, P., Hetmański, T., and Skórka, P. (2018). Experimentally evoked same-sex sexual behaviour in pigeons: Better to be in a female-female pair than alone. Sci. Rep. 8(1), 1–7. doi: 10.1038/s41598-018-20128-3.
38. Kitamoto, T. (2002). Conditional disruption of synaptic transmission induces male-male courtship behavior in Drosophila. Proc. Natl. Acad. Sci. U. S. A. *99*, 13232–13237.
39. Krstic, D., Boll, W., and Noll, M. (2013). Influence of the White locus on the courtship behavior of Drosophila males. PLoS One *8*, e77904.
40. Kuriwada, T. (2017). Male–male courtship behaviour, not relatedness, affects the intensity of contest competition in the field cricket. Anim. Behav. 126, 217–220. doi: 10.1016/j.anbehav.2017.02.009
41. Lacaille, F., Hiroi, M., Twele, R., Inoshita, T., Umemoto, D., Manière, G., Marion-Poll, F., Ozaki, M., Francke, W., Cobb, M., *et al.* (2007). An inhibitory sex pheromone tastes bitter for Drosophila males. PLoS One *2*, 1–7.
42. Lane, S.M., Haughan, A.E., Evans, D., Tregenza, T., and House, C.M. (2016). Same-sex sexual behaviour as a dominance display. Anim. Behav. *114*, 113–118.
43. Liu, T., Dartevelle, L., Yuan, C., Wei, H., Wang, Y., Ferveur, J.-F., and Guo, A. (2008). Increased dopamine level enhances male-male courtship in Drosophila. J. Neurosci. *28*, 5539–5546.
44. Liu, T., Dartevelle, L., Yuan, C., Wei, H., Wang, Y., Ferveur, J.F., and Guo, A. (2009). Reduction of dopamine level enhances the attractiveness of male Drosophila to other males. PLoS One *4*, 1–5.
45. Liu, T., Wang, Y., Tian, Y., Zhang, J., Zhao, J., and Guo, A. (2020). The receptor channel formed by ppk25, ppk29 and ppk23 can sense the Drosophila female pheromone 7,11-heptacosadiene. Genes Brain Behav. 19(2), 1–10. doi: 10.1111/gbb.12529.
46. Liu, Z., Li, X., Prasifka, J.R., Jurenka, R., and Bonning, B.C. (2008). Overexpression of Drosophila juvenile hormone esterase binding protein results in anti-JH effects and reduced pheromone abundance. Gen. Comp. Endocrinol. *156*, 164–172.
47. Logue, D.M., Mishra, S., McCaffrey, D., Ball, D., and Cade, W.H. (2009). A behavioral syndrome linking courtship behavior toward males and females predicts reproductive success from a single mating in the hissing cockroach, *Gromphadorhina portentosa*. Behav. Ecol. *20*, 781–788.
48. Macchiano, A., Razik, I., and Sagot, M. (2018). Same-sex courtship behaviors in male-biased populations: evidence for the mistaken identity hypothesis. Acta Ethol. 21(3), 147–151. doi: 10.1007/s10211-018-0293-8.
49. MacFarlane, G.R., Blomberg, S.P., and Vasey, P.L. (2010). Homosexual behaviour in birds: frequency of expression is related to parental care disparity between the sexes. Anim. Behav. *80*, 375–390.
50. MacFarlane, G.R., Blomberg, S.P., Kaplan, G., and Rogers, L.J. (2007). Same-sex sexual behavior in birds: Expression is related to social mating system and state of development at hatching. Behav. Ecol. *18*, 21–33.
51. Meissner, G.W., Manoli, D.S., Chavez, J.F., Knapp, J.-M., Lin, T.L., Stevens, R.J., Mellert, D.J., Tran, D.H., and Baker, B.S. (2011). Functional dissection of the neural substrates for sexual behaviors in *Drosophila melanogaster*. Genetics *189*, 195–211.
52. Monk, J. D., Giglio, E., Kamath, A., Lambert, M. R., and McDonough, C. E. (2019). An alternative hypothesis for the evolution of same-sex sexual behaviour in animals. Nat. Ecol. Evol. 3(12), 1622–1631. doi: 10.1038/s41559-019-1019-7.
53. Murakami, S., and Itoh, M.T. (2003). Removal of both antennae influences the courtship and aggressive behaviors in male crickets. J. Neurobiol. *57*, 110–118.
54. Oliveira, R.F., and Almada, V.C. (1998). Mating tactics and male-male courtship in the lek-breeding cichlid *Oreochromis mossambicus*. J. Fish *52*, 1115–1129.
55. Pincemy, G., Dobson, F.S., and Jouventin, P. (2010). Homosexual mating displays in penguins. Ethology *116*, 1210–1216.
56. Pivnick, K.A., Lavoir-Dornik, J., and McNeil, J. (1992). The role of the androconia in the mating behaviour of the European skipper, *Thymelicus lineola*, and evidence for a male sex pheromone. Physiol. Entomol. *17*, 260–268.
57. Rayner, J. G., and Bailey, N. W. (2019). Testing the role of same-sex sexual behaviour in the evolution of alternative male reproductive phenotypes. Anim. Behav. 157, 5–11. doi: 10.1016/j.anbehav.2019.08.017.
58. Riccucci, M. (2011). Same-sex sexual behaviour in bats. Hystrix *22*, 139–147.
59. Scharf, I., and Martin, O.Y. (2013). Same-sex sexual behavior in insects and arachnids: prevalence, causes, and consequences. Behav. Ecol. Sociobiol. *67*, 1719–1730.
60. Sellami, A., Wegener, C., and Veenstra, J.A. (2012). Functional significance of the copper transporter ATP7 in peptidergic neurons and endocrine cells in *Drosophila melanogaster*. FEBS Lett. *586*, 3633–3638.
61. Shine, R., Harlow, P., LeMaster, M., Moore, I., and Mason, R. (2000). The transvestite serpent: why do male garter snakes court (some) other males? Anim. Behav. *59*, 349–359.
62. Silva, V., Palacios-Muñoz, A., Volonté, M., Frenkel, L., Ewer, J., and Ons, S. (2021). Orcokinin neuropeptides regulate reproduction in the fruit fly, *Drosophila melanogaster*. Insect Biochem Mol. 139. doi: 10.1016/j.ibmb.2021.103676.
63. Skinner, B. (2016). Mathematical toy model inspired by the problem of the adaptive origins of the sexual orientation continuum. R. Soc. Open Sci. 3(9). doi: 10.1098/rsos.160403.
64. Songvorawit, N., Butcher, B. A., and Chaisuekul, C. (2019). Size does not matter: same-sex sexual behavior occurred regardless of mandible size in male stag beetle *Aegus chelifer chelifer* (Coleoptera: Lucanidae). J Insect Behav. 32(4–6), 282–289. doi: 10.1007/s10905-019-09733-w.
65. Toda, H., Zhao, X., and Dickson, B.J. (2012). The Drosophila female aphrodisiac pheromone activates ppk23+ sensory neurons to elicit male courtship behavior. Cell Rep. *1*, 599–607.
66. Ungerfeld, R., Giriboni, J., Freitas-de-Melo, A., and Lacuesta, L. (2014). Homosexual behavior in male goats is more frequent during breeding season and in bucks isolated from females. Horm. Behav. *65*, 516–520.
67. Ungerfeld, R., Ramos, M.A., and Bielli, A. (2007). Relationship between male-male and male-female sexual behavior in 5-6-month-old male lambs. Anim. Reprod. Sci. *100*, 385–390.
68. Vijayan, V., Thistle, R., Liu, T., Starostina, E., and Pikielny, C.W. (2014). Drosophila pheromone-sensing neurons expressing the ppk25 ion channel subunit stimulate male courtship and female receptivity. PLoS Genet. *10*.
69. Wang, K., Guo, Y., Wang, F., and Wang, Z. (2011). Drosophila TRPA channel painless inhibits male-male courtship behavior through modulating olfactory sensation. PLoS One *6*.
70. Wang, L., Han, X., Mehren, J., Hiroi, M., Billeter, J.-C., Miyamoto, T., Amrein, H., Levine, J.D., and Anderson, D.J. (2011b). Hierarchical chemosensory regulation of male-male social interactions in Drosophila. Nat. Neurosci. *14*, 757–762.
71. Wang, Q., Chen, L., Li, J., and Yin, X. (1996). Mating behavior of *Phytoecia rufiventris* Gautier (Coleoptera: Cerambycidae). J. Insect Behav. *9*, 47–60.
72. Weng, R., Chin, J.S.R., Yew, J.Y., Bushati, N., and Cohen, S.M. (2013). miR-124 controls male reproductive success in Drosophila. Elife *2013*, 1–16.
73. Xia, R. Y., Li, M. Q., Wu, Y. S., Qi, Y. X., Ye, G. Y., and Huang, J. (2016). A new family of insect muscarinic acetylcholine receptors. Insect Mol Biol. 25(4), 362–369. doi: 10.1111/imb.12229.
74. Yamamoto, S., and Seto, E.S. (2014). Dopamine dynamics and signaling in Drosophila: an overview of genes, drugs and behavioral paradigms. Exp. Anim. *63*, 107–119.
75. Zhan, H., Li, D., Dewer, Y., Niu, C., Li, F., and Luo, C. (2021). Identification and functional characterization of odorant-binding proteins 69a and 76a of *Drosophila suzukii*. Heliyon. 7(3), e06427. doi: 10.1016/j.heliyon.2021.e06427.
76. Zhang, S.D., Odenwald, W.F., Odenwald, F., Zhang, S.D., Odenwald, W.F., Odenwald, F., Zhang, S.D., and Odenwald, W.F. (1995). Misexpression of the white (w) gene triggers male-male courtship in Drosophila. Proc. Natl. Acad. Sci. U. S. A. *92*, 5525–5529.

## References S2. Systematic review of eavesdropping, exploitation and audience effect during male-female courtship display interactions.

1. Benelli, G., Daane, K.M., Canale, A., Niu, C.Y., Messing, R.H., and Vargas, R.I. (2014). Sexual communication and related behaviours in Tephritidae: current knowledge and potential applications for Integrated Pest Management. J. Pest Sci. (2004). *87*, 385–405. doi: 10.1007/s10340-014-0577-3
2. Benelli, G., Donati, E., Romano, D., Ragni, G., Bonsignori, G., Stefanini, C., and Canale, A. (2016). Is bigger better? Male body size affects wing-borne courtship signals and mating success in the olive fruit fly, *Bactrocera oleae* (Diptera: Tephritidae). Insect Sci. *23*, 869–880. doi: 10.1111/1744-7917.12253
3. Borgia, G., and Mueller, U. (1992). Bower destruction, decoration stealing and female choice in the spotted bowerbird *Chlamydera maculata*. EMU *92*, 11–18. doi: 10.1071/MU9920011
4. Boutin, S.R.T., Harrison, S.J., Fitzsimmons, L.P., McAuley, E.M., and Bertram, S.M. (2016). Same-sex sexual behaviour in crickets: understanding the paradox. Anim. Behav. *114*, 101–110. doi: 10.1016/j.anbehav.2016.01.022
5. Churchill, E. R., Bridle, J. R., & Thom, M. D. F. (2020). Spatially clustered resources increase male aggregation and mating duration in *Drosophila melanogaster*. Anim. Behav. 169, 45–50. doi: 10.1016/j.anbehav.2020.09.002
6. Dunn, A.M., and Zann, R.A. (1997). Effects of pair bond and presence of conspecifics on singing in captive zebra finches. Behavior *134*, 127–142. doi: 10.1163/156853997X00313
7. Friesen, C. R., Powers, D. R., & Mason, R. T. (2017). Using whole-group metabolic rate and behaviour to assess the energetics of courtship in red-sided garter snakes. Anim. Behav. 130, 177–185. doi: 10.1016/j.anbehav.2017.06.020
8. Fritz, W. F., Sena, L. S., Becker, S. E., & Katz, L. S. (2019). Differential effects of androgens, estrogens and socio-sexual context on sexual behaviors in the castrated male goat. Horm. Behav. 109, 10–17. doi: 10.1016/j.yhbeh.2019.01.008
9. Gleeson, D.J. (2006). Context-dependent effect of social environment on immune response and sexual signalling in male zebra finches. Aust. J. Zool. *54*, 375–379. doi: 10.1071/ZO06001
10. Gobbi, P. C., Nunes, A. M., Zefa, E., & Garcia, F. R. M. (2020). Influence of wing bands and behavior of *Anastrepha fraterculus* (Diptera: Tephritidae) by the presence of *Megafreya sutrix* (Araneae: Salticidae). An. Acad. Brasil. Cienc. 92, 1–10. doi: 10.1590/0001-3765202020181306
11. Hendrichs, J., Katsoyannos, B.I., Wornoayporn, V., and Hendrichs, M.A. (1994). Odour-mediated foraging by yellowjacket wasps (Hymenoptera: Vespidae): predation on leks of pheromone-calling Mediterranean fruit fly males (Diptera: Tephritidae). Oecologia *99*, 88–94. doi: 10.1007/BF00317087
12. Himuro, C., Hosokawa, T., and Suzuki, N. (2006). Alternative mating strategy of small male *Megacopta punctatissima* (Hemiptera: Plataspidae) in the presence of large intraspecific males. Ann. Entomol. Soc. Am. *99*, 974–977.
13. Ishikawa, Y., Okamoto, N., Yoneyama, Y., Maeda, N., & Kamikouchi, A. (2019). A single male auditory response test to quantify auditory behavioral responses in *Drosophila melanogaster*. J. of Neurogenet. 33(2), 64–74. doi: 10.1080/01677063.2019.1611805
14. Jarvis, E.D., Scharff, C., Grossman, M.R., Ramos, J.A., and Nottebohm, F. (1998). For whom the bird sings. Neuron *21*, 775–788. doi: 10.1016/S0896-6273(00)80594-2
15. Jesse, F., and Riebel, K. (2012). Social facilitation of male song by male and female conspecifics in the zebra finch, *Taeniopygia guttata*. Behav. Processes *91*, 262–266. doi: 10.1016/j.beproc.2012.09.006
16. Kumano, N., Kuriwada, T., Shiromoto, K., Haraguchi, D., & Kohama, T. (2010). Effect of body size and sex ratio on male alternative mating tactics of the West Indian sweetpotato weevil, *Euscepes postfasciatus*. Entomol. Exp. Appl. 135(2), 154–161. doi: 10.1111/j.1570-7458.2010.00975.x
17. Lacuesta, L., and Ungerfeld, R. (2012). Sexual performance and stress response of previously unknown rams after grouping them in dyads. Anim. Reprod. Sci. *134*, 158–163. doi: 10.1016/j.anireprosci.2012.08.021
18. Lane, S.M., Solino, J.H., Mitchell, C., Blount, J.D., Okada, K., Hunt, J., and House, C.M. (2015). Rival male chemical cues evoke changes in male pre- and post-copulatory investment in a flour beetle. Behav. Ecol. *26*, 1021–1029. doi: 10.1093/beheco/arv047
19. Lin, L.C., Vanier, D.R., and London, S.E. (2014). Social information embedded in vocalizations induces neurogenomic and behavioral responses. PLoS One *9*. doi: 10.1371/journal.pone.0112905
20. Maguire, C.P., Lizé, A., and Price, T.A.R. (2015). Assessment of rival males through the use of multiple sensory cues in the fruitfly *Drosophila pseudoobscura*. PLoS One *10*, 1–13. doi: 10.1371/journal.pone.0123058
21. Martin, J. O., & Burley, N. T. (2021). Elucidating mutual mate choice: effects of trial design on preferences of male zebra finches. Behav. Ecol. 32(6), 1306–1320. doi: 10.1093/beheco/arab097
22. Nelson, C.M. (1995). Male size, spawning pit size and female mate choice in a lekking cichlid fish. Anim. Behav. *50*, 1587–1599. doi: 10.1016/0003-3472(95)80013-1
23. Ng, S.H., Shankar, S., Shikichi, Y., Akasaka, K., Mori, K., and Yew, J.Y. (2014). Pheromone evolution and sexual behavior in Drosophila are shaped by male sensory exploitation of other males. Proc. Natl. Acad. Sci. *111*, 3056–3061. doi: 10.1073/pnas.1313615111
24. Noh, S., and Henry, C.S. (2015). Within-species mate preferences do not contribute to the maintenance of sexually monomorphic mating signals in green lacewings. Ethology *121*, 714–724. doi: 10.1111/eth.12385
25. Omena Junior, R.S. (2009). Behavior of the Guianan Cock-of-the-rock *Rupicola rupicola* Cotingidae in Presidente Figueiredo, Amazonas, Brazil. Rev. Bras. Ornitol. *17*, 87–95.
26. Papanastasiou, S.A., Diamantidis, A.D., Nakas, C.T., Carey, J.R., and Papadopoulos, N.T. (2011). Dual reproductive cost of aging in male medflies: dramatic decrease in mating competitiveness and gradual reduction in mating performance. J. Insect Physiol. *57*, 1368–1374. doi: 10.1016/j.jinsphys.2011.07.004
27. Price, E.O., Borgwardt, R., Orihuela, A., and Dally, M.R. (1998). Sexual stimulation in male sheep and goats. Appl. Anim. Behav. Sci. *59*, 317–322. doi: 10.1016/S0168-1591(98)00112-9
28. Price, E.O., Wallach, S.J.R., and Dally, M.R. (1991). Effects of sexual stimulation on the sexual performance of rams. Appl. Anim. Behav. Sci. *30*, 330–340. doi: 10.1016/0168-1591(91)90138-N
29. Reichard, D.G., and Anderson, R.C. (2015). Why signal softly? The structure, function and evolutionary significance of low-amplitude signals. Anim. Behav. *105*, 253–265. doi: 10.1016/j.anbehav.2015.04.017
30. Reynolds, S.M., Christman, M.C., Uy, J.A.C., Patricelli, G.L., Braun, M.J., and Borgia, G. (2009). Lekking satin bowerbird males aggregate with relatives to mitigate aggression. Behav. Ecol. *20*, 410–415. doi: 10.1093/beheco/arn146
31. Rouse, J., and Bretman, A. (2016). Exposure time to rivals and sensory cues affect how quickly males respond to changes in sperm competition threat. Anim. Behav. *122*, 1–8. doi: 10.1016/j.anbehav.2016.09.011
32. Schöneich, S. (2020). Neuroethology of acoustic communication in field

crickets – from signal generation to song recognition in an insect brain. Prog. Neurob. 194, 101882. doi: 10.1016/j.pneurobio.2020.101882

1. Shelly, T. (2000). Male signalling and lek attractiveness in the Mediterranean fruit fly. Anim. Behav. *60*, 245–251. doi: 10.1006/anbe.2000.1470
2. Shine, R., Langkilde, T., Wall, M., and Mason, R.T. (2005a). Alternative male mating tactics in garter snakes, *Thamnophis sirtalis parietalis*. Anim. Behav. *70*, 387–396. doi: 10.1016/j.anbehav.2005.04.001
3. Shine, R., O’Donnell, R.P., Langkilde, T., Wall, M.D., and Mason, R.T. (2005b). Snakes in search of sex: the relation between mate-locating ability and mating success in male garter snakes. Anim. Behav. *69*, 1251–1258. doi: 10.1016/j.anbehav.2004.10.005
4. Thornhill, R. (1980). Mate choice in *Hylobittacus apicalis* (Insecta: Mecoptera) and its relation to some models of female choice. Evolution (N. Y). *34*, 519. doi: 10.2307/2408221
5. Tinghitella, R.M., and Zuk, M. (2009). Asymmetric mating preferences accommodated the rapid evolutionary loss of a sexual signal. Evolution (N. Y). *63*, 2087–2098. doi: 10.1111/j.1558-5646.2009.00698.x
6. Trail, P.W. (1990). Why should lek-breeders be monomorphic? Evolution (N. Y). *44*, 1837. doi: 10.1111/j.1558-5646.1990.tb05254.x
7. Ungerfeld, R. (2012). Sexual behavior of medium-ranked rams toward non-estrual ewes is stimulated by the presence of low-ranked rams. J. Vet. Behav. Clin. Appl. Res. *7*, 84–87. doi: 10.1016/j.jveb.2011.05.023
8. Ungerfeld, R., and González-Pensado, S.P. (2009). Social dominance and courtship and mating behaviour in rams in non-competitive and competitive pen tests. Reprod. Domest. Anim. *44*, 44–47. doi: 10.1111/j.1439-0531.2007.00987.x
9. Ungerfeld, R., and Lacuesta, L. (2015). Competition between different social ranked rams has similar effects on testosterone and sexual behaviour throughout the year. Reprod. Domest. Anim. *50*, 1022–1027. doi: 10.1111/rda.12630
10. Ungerfeld, R., Orihuela, A., & Pérez-Clariget, R. (2019). Sexual behavior of subordinate, but not dominant, rams increases following observed sexual activity. Theriogenology. 129, 99–102. doi: 10.1016/j.theriogenology.2019.02.033
11. Ungerfeld, R., Ramos, M.A., and Bielli, A. (2007). Relationship between male-male and male-female sexual behavior in 5-6-month-old male lambs. Anim. Reprod. Sci. *100*, 385–390. doi: 10.1016/j.anireprosci.2006.09.022
12. Vignal, C., Mathevon, N., and Mottin, S. (2004). Audience drives male songbird response to partner’s voice. Nature *430*, 448–451. doi: 10.1038/nature02645
13. Wackermannová, M., Horký, P., Amorim, M.C.P., and Fonseca, P.J. (2017). Computer manipulated stimuli as a research tool in Mozambique tilapia *Oreochromis mossambicus*. Acta Ethol. *20*, 85–94. doi: 10.1007/s10211-017-0252-9
