# Supplementary material for: The role of intrasexual competition on the evolution of male-male courtship display: a systematic review
Source: PeerJ. 2023 Feb 2;10:e14638. doi: 10.7717/peerj.14638 (PMC9899439; doi:10.7717/peerj.14638)
Supplement: Supplemental Information 7 [file peerj-11-14638-s007.docx]

**Rationale beyond the systematic review**

If male courtship display (either a courtship song, dance or any other kind of ritualized behaviour) has a dual function, when directed to other males it will be qualified as same-sex sexual behaviour. Complementarily, when a male-male courtship display is described as same-sex sexual behaviour in an intrasexual competition context, a dual function hypothesis for the courtship display may well be equated. Surprisingly, despite their complementary conceptual approaches, these two research fields have been working separately.

**Study contribution**

By systematically reviewing, first, studies reporting male-male courtship display in non-human animals and suggested hypotheses, and, second, studies reporting interaction with competitor males during male-female courtship display, we found evidence supporting a competition-driven male-male courtship display as a result of bystander pressure. In this sense, our study goes beyond general review studies about same-sex sexual behaviours and gives a novel perspective to be explored of a link between sexual signal dual function and same-sex sexual behaviour.
